# Widespread dissemination of the RepA_N-type megaplasmid conferring a carbohydrate utilization advantage in clonal complex 17 *Enterococcus faecium* in Taiwan

**DOI:** 10.1128/spectrum.03793-25

**Published:** 2026-07-14

**Authors:** Yi-Jing Chen, Hsiao-Ching Peng, Dong-Ying Wu, Shang-Yi Lin, Fang-Yi Li, Yu-Ting Wang, Chung-Yu Chang, Wei-Chun Hung

**Affiliations:** 1 School of Medicine, College of Medicine, Kaohsiung Medical University 38023, Kaohsiung, Taiwan; 2 Department of Microbiology and Immunology, College of Medicine, Kaohsiung Medical University 38023, Kaohsiung, Taiwan; 3 Department of Public Health, College of Health Science, Kaohsiung Medical University 38023, Kaohsiung, Taiwan; 4 Division of Infectious Diseases, Department of Internal Medicine, Kaohsiung Medical University Hospital 89234, Kaohsiung, Taiwan; 5 Division of Research and Analysis, Food and Drug Administration, Ministry of Health and Welfare 63123, Taipei, Taiwan; 6 School of Post-Baccalaureate Medicine, College of Medicine, Kaohsiung Medical University 38023, Kaohsiung, Taiwan; Taichung Veterans General Hospital 40293, Taichung, Taiwan

**Keywords:** *Enterococcus faecium*, megaplasmid, carbohydrate utilization, gentamicin, *aac(6′)-Ie-aph(2″)-Ia*

## Abstract

*Enterococcus faecium* has become a major nosocomial pathogen, primarily driven by its ability to acquire mobile genetic elements carrying antimicrobial resistance or metabolic traits. Our previous study identified a megaplasmid conferring a high-level gentamicin resistance (HLGR), which can undergo recombination between intact and IS*1216V*-disrupted *aac(6′)-Ie-aph(2″)-Ia* genes, resulting in a non-HLGR phenotype. In this study, we further characterized the RepA_N-type megaplasmid in sequence type (ST)17 and ST78 *E. faecium* and examined its distribution among 465 isolates collected between 2011 and 2020 in Taiwan. Both sublineages carried gene clusters associated with carbohydrate utilization, albeit with differences in repertoires. Loss of the RepA_N megaplasmid in ST17 isolate E37 impaired growth on sucrose, confirming its contribution to carbohydrate metabolism. Screening of clinical isolates showed that *repA_N* and *scrB* (sucrose utilization) genes were highly prevalent (90.8% and 85.8%, respectively) in the 10 years, whereas IS*1216V*-inserted *aac(6′)-Ie-aph(2″)-Ia* decreased over time (84.6% in 2011 and 55.7% in 2020), paralleling a decline in HLGR rates (57.7% in 2011 and 45.9% in 2020). Among the 465 *E. faecium* isolates, ST17 and ST78, both belonging to clonal complex 17, were the most dominant sublineages. The expansion of ST78 vancomycin-resistant *E. faecium* since 2015 corresponded with the emergence of type III IS*1216V*-inserted *aac(6′)-Ie-aph(2″)-Ia*, while ST17, mainly carrying type II IS*1216V* insertion, remained stable throughout the decade. Collectively, these results indicate that the RepA_N-type megaplasmid was widely disseminated and may enhance metabolic adaptability in *E. faecium*, supporting its persistence in clinical environments.

**IMPORTANCE:**

*Enterococcus faecium* is a major hospital-associated pathogen with high genomic plasticity. This study characterized a widespread RepA_N-type megaplasmid, which conferred both high-level gentamicin resistance and enhanced carbohydrate utilization. The co-selection of antimicrobial resistance and metabolic adaptability on a single mobile genetic element may underlie the evolutionary success of dominant clinical lineages such as sequence type (ST)17 and ST78 in Taiwan. These findings provide a framework for understanding how plasmids contribute to the persistence and spread of *E. faecium* in healthcare settings.

## INTRODUCTION

Once regarded as a benign intestinal commensal, vancomycin-resistant *Enterococcus faecium* (VREfm) has been designated a WHO bacterial priority pathogen (1). Its remarkable genomic plasticity enables adaptation to hospital environments, primarily through the acquisition of multiple antibiotic resistance determinants (2–5). The clonal complex 17 (CC17) lineage, in particular, is a highly hospital-adapted lineage responsible for the increasing global spread of vancomycin resistance (6).

In addition to antimicrobial resistance, carbohydrate metabolism plays a critical role in the survival and proliferation of *E. faecium* during colonization and infection (7–14). Phosphotransferase systems (PTS), which mediate the uptake and utilization of specific carbohydrates, play a crucial role in adaptation in enterococci. A chromosomally encoded PTS^clin^ enriched in clinical isolates has been shown to facilitate intestinal colonization during antibiotic treatment (8). Furthermore, the acquisition of a novel linear plasmid in VREfm enables the utilization of N-acetyl-galactosamine, a key component of gut mucins, thereby enhancing intestinal colonization (10).

Our previous work demonstrated that *E. faecium* can alter its high-level gentamicin resistance (HLGR) phenotype through recombination events between an intact and an IS*1216V*-inserted *aac(6′)-Ie-aph(2″)-Ia* gene, resulting in the irreversible loss of resistance (15). We identified two major IS*1216V* insertion types, type II (at 129 nt of *aac(6′)-Ie-aph(2″)-Ia* gene) and type III (at 337 nt). In the current study, we identified that the gentamicin resistance gene *aac(6′)-Ie-aph(2″)-Ia* was located on a RepA_N plasmid (170–273 kb) that harbored multiple carbohydrate utilization gene clusters, consistent with the size range commonly defined for megaplasmids in enterococci (7). Notably, loss of this megaplasmid impaired growth on sucrose, implying the co-selection of metabolic and resistance traits. Longitudinal analysis of bloodstream isolates revealed that this megaplasmid has persisted within the CC17 lineage over a decade, suggesting its potential contribution to the lineage’s success in clinical settings.

## RESULTS

### A RepA_N megaplasmid in the ST17 *E. faecium* strain E37 carried type II IS*1216V*-inserted *aac(6′)-Ie-aph(2″)-Ia* gene and multiple carbohydrate utilization gene clusters

**Fig 1:**
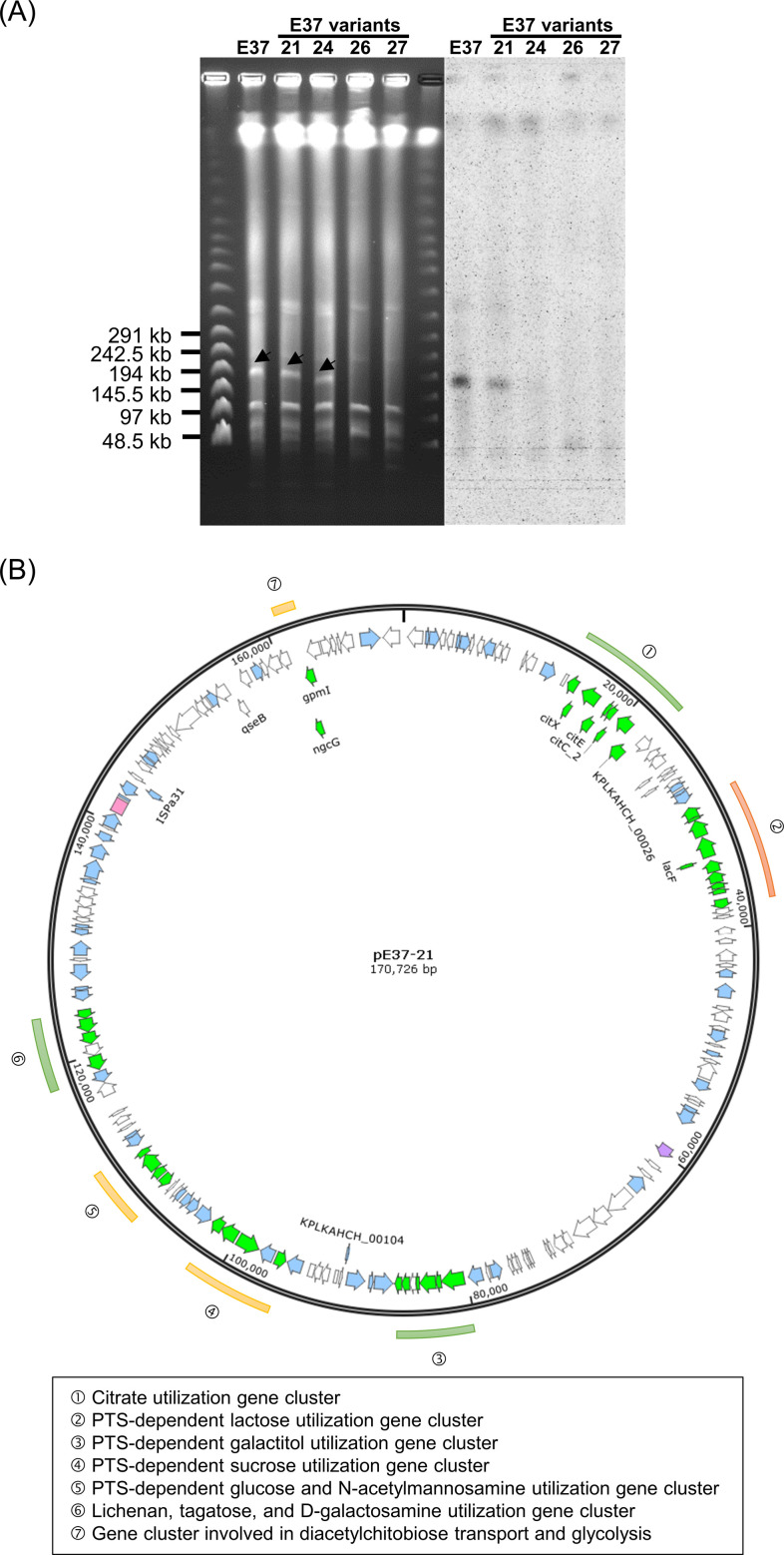
The RepA_N-type megaplasmid in ST17 *E. faecium* strain E37 with HLGR phenotype and its variants with non-HLGR phenotype. (**A**) S1-nuclease PFGE analysis followed by Southern blot hybridization using an *aac(6′)-Ie-aph(2″)-Ia* probe. The size standard is a 48.5 kb ladder. The black arrow indicates the megaplasmid carrying *aac(6′)-Ie-aph(2″)-Ia*. (**B**) Genetic map of the RepA_N-type megaplasmid in E37-21. The inner circle consists of open reading frames (arrows) and truncated genes (rectangles), with colors indicating gene functions as follows: blue, insertion sequences (ISs); green, gene clusters involved in the transport and utilization of carbohydrates or related compounds; purple, replication initiator gene (*repA_N*); pink, *aac(6′)-Ie-aph(2″)-Ia*. The outer circle indicates the comparative distribution of carbohydrate utilization gene clusters, with colors as follows: green, gene clusters found in ST17 *E. faecium* but not in ST78; yellow, gene clusters found in both ST17 and ST78 *E. faecium*; orange, gene clusters found in both ST17 and ST78 *E. faecium* that are highly divergent at the nucleotide level.

Our previous study demonstrated that the sequence type (ST)17 *E. faecium* strain E37 with HLGR phenotype harbored both an intact and a type II IS*1216V*-inserted *aac(6′)-Ie-aph(2″)-Ia* gene (15). The stability assay generated a series of E37 variants that transitioned from an HLGR to a non-HLGR phenotype. PCR mapping revealed that the variant E37-21 still carried a type II IS*1216V*-inserted *aac(6′)-Ie-aph(2″)-Ia* gene, while E37-24, E37-26, and E37-27 were negative for *aac(6′)-Ie-aph(2″)-Ia* gene. Notably, the IS*1216V* element in the type II insertion in E37-21 was truncated, as previously reported (15). S1-nuclease PFGE analysis, followed by Southern blot hybridization using an *aac(6′)-Ie-aph(2″)-Ia* probe, showed that E37-24 harbored a plasmid of reduced size and had lost the *aac(6′)-Ie-aph(2″)-Ia* gene compared to E37-21. In contrast, E37-26 and E37-27 had lost the entire plasmid (Fig. 1A).

**Fig 2:**
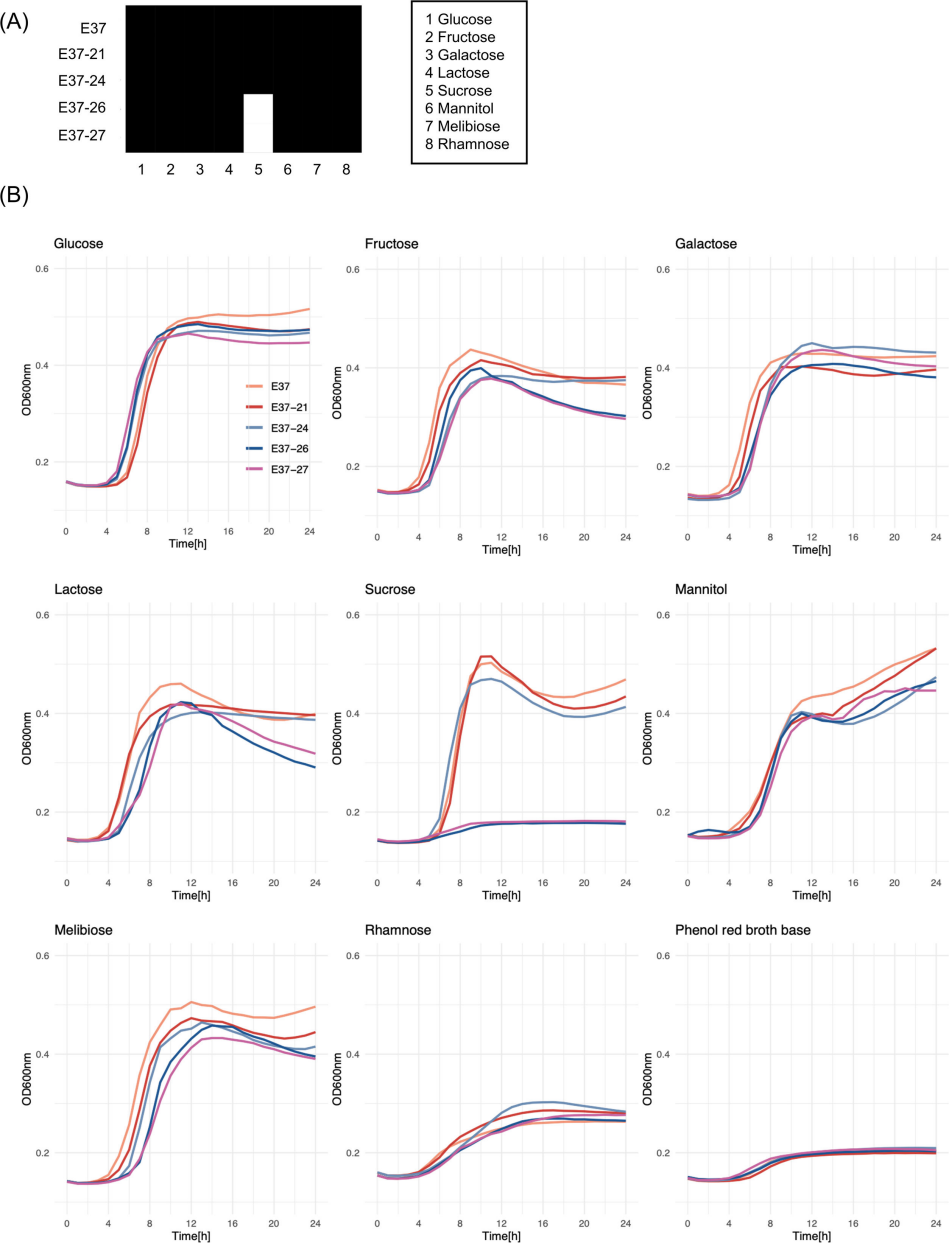
Growth of E37 and its variants in phenol red broth or broth supplemented with various carbon sources. (**A**) Summary of carbohydrate utilization ability based on growth curves. Black indicates the ability to utilize the indicated carbohydrate; white indicates inability. (**B**) Growth curves in phenol red broth alone or supplemented with the indicated carbon sources.

Sequence analysis revealed that the IS*1216V*-inserted *aac(6′)-Ie-aph(2″)-Ia* in E37-21 was located on a RepA_N-type plasmid, pE37-21, with a total size of 170,726 nt (Fig. 1B). The pE37-21 contained various ISs and multiple genes predicted to be involved in the transport, utilization, and metabolism of carbohydrates (including diacetylchitobiose, glucose, lactose, lichenan, sucrose, and tagatose) or related compounds (including N-acetylmannosamine, citrate, galactitol, and D-galactosamine), as shown in Fig. 1B and Table S1. Fermentation assays demonstrated that variants lacking the megaplasmid (E37-26 and E37-27) failed to utilize sucrose. In the growth curves, a decrease in OD_600_ after 12–24 h of incubation was observed in E37-26 and E37-27 under fructose- and lactose-supplemented conditions, which may reflect nutrient depletion or the accumulation of metabolic byproducts affecting cell viability or optical density. Carbohydrate utilization for other substrates remained unchanged (Fig. 2).

### The RepA_N megaplasmid in ST78 *E. faecium* with type III IS*1216V*-inserted *aac(6′)-Ie-aph(2″)-Ia* gene and multiple carbohydrate utilization gene clusters

**Fig 3:**
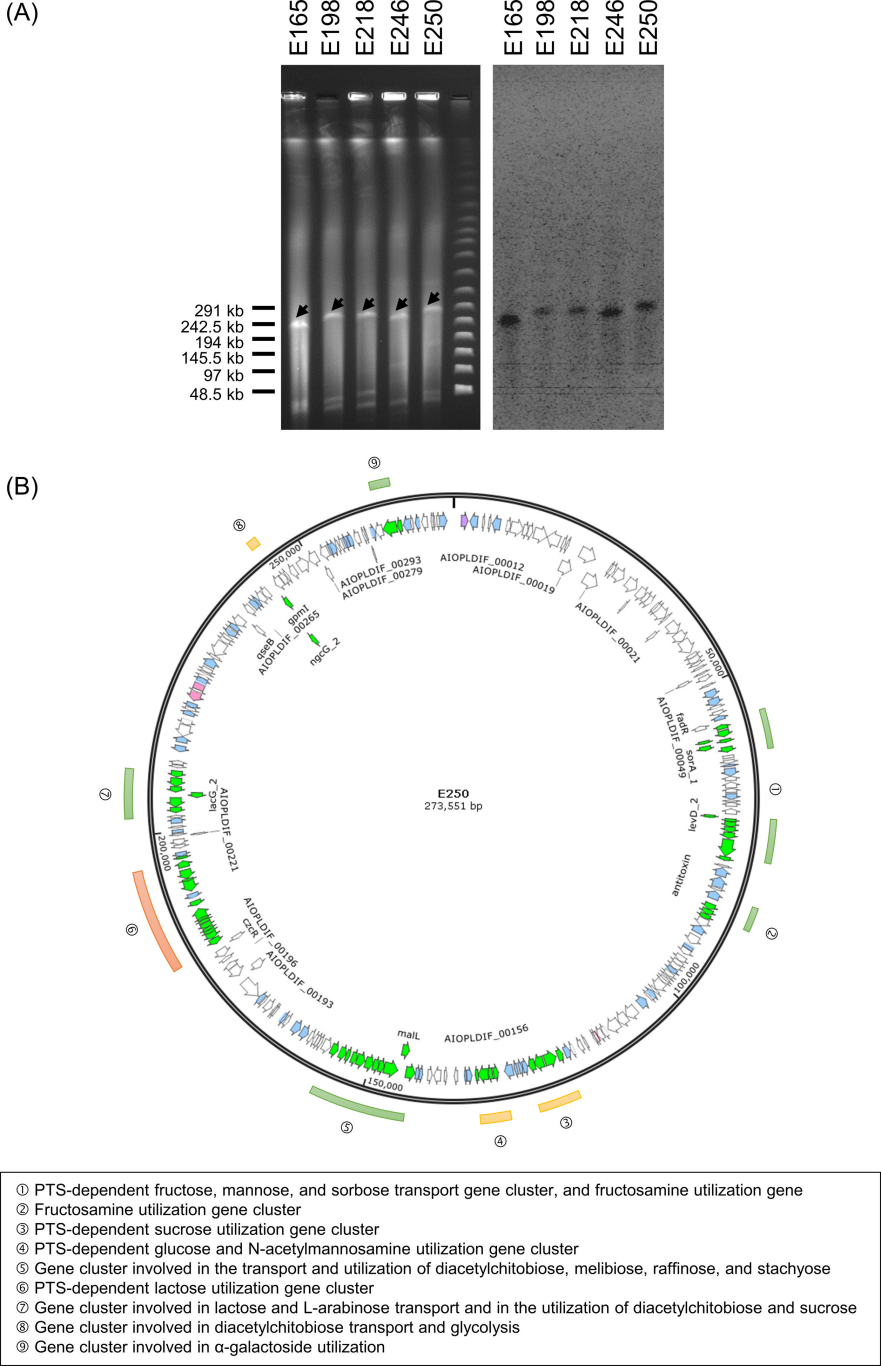
The RepA_N-type megaplasmid in ST78 *E. faecium*. (**A**) S1-nuclease PFGE analysis followed by Southern blot hybridization using an *aac(6′)-Ie-aph(2″)-Ia* probe. The size standard is a 48.5 kb ladder. The black arrow indicates the megaplasmid carrying *aac(6′)-Ie-aph(2″)-Ia*. (**B**) Genetic map of the RepA_N-type megaplasmid in E250. The inner circle consists of open reading frames (arrows) and truncated genes (rectangles), with colors indicating gene functions as follows: blue, ISs; green, gene clusters involved in the transport and utilization of carbohydrates or related compounds; purple, replication initiator gene; pink, *aac(6′)-Ie-aph(2″)-Ia*. The outer circle indicates the comparative distribution of carbohydrate utilization gene clusters, with colors as follows: green, gene clusters found in ST78 *E. faecium* but not in ST17; yellow, gene clusters found in both ST17 and ST78 *E. faecium*; orange, gene clusters found in both ST17 and ST78 *E. faecium* that are highly divergent at the nucleotide level.

ST17 and ST78 are the major genotypes of VREfm in Taiwan. Our previous study identified ST78 *E. faecium,* carrying a type III IS*1216V* insertion within the *aac(6′)-Ie-aph(2″)-Ia* gene (15). In this study, we further characterized five ST78 *E. faecium* isolates, selected to represent the genetic diversity of type III IS*1216V* insertion-carrying strains, by S1-nuclease PFGE analysis. The *aac(6′)-Ie-aph(2*″*)-Ia* gene was located on megaplasmids ranging from approximately 242.5 to 291 kb (Fig. 3A), larger than the RepA_N megaplasmid identified in ST17 (170,726 bp; Fig. 1).

E198 (isolated in 2016; non-HLGR phenotype) and E250 (isolated in 2015; HLGR phenotype) were selected for plasmid sequencing. The plasmids pE198 and pE250 measured 272,849 and 273,351 nt, respectively, sharing a 268,552 nt homologous region (98.3% identity). Significant differences included insertions of IS*Efa11* and IS*Spn10* in pE198, an IS*16* insertion in pE250, and the presence of an intact *aac(6′)-Ie-aph(2″)-Ia* in pE250 (summarized in Table S2). No variations were observed in carbohydrate utilization gene clusters.

The plasmid map of pE250 is shown in Fig. 3B. Nine gene clusters related to carbohydrate transport and metabolism were identified (summarized in Table S3). The PTS-dependent sucrose utilization gene cluster, the PTS-dependent glucose and N-acetylmannosamine utilization gene cluster, and the gene cluster involved in diacetylchitobiose transport and glycolysis were conserved in both pE250 and pE37-21 (Fig. 1B).

Further comparison between pE37-21 and pE250 is shown in Fig. S1. The two plasmids shared approximately 108,480 bp of homologous regions with an overall nucleotide identity of 96.9%, accounting for 63.5% of pE37-21 and 39.7% of pE250, respectively. Notably, the PTS-dependent lactose utilization gene cluster was present in both pE37-21 and pE250, although the nucleotide sequences were highly divergent, showing less than 70% similarity. The homologous regions were distributed across multiple conserved segments throughout the plasmids, several of which corresponded to three gene clusters involved in carbohydrate transport and utilization. These findings suggested that RepA_N-type megaplasmids shared conserved genetic features but exhibited structural diversity, rather than representing a single highly conserved plasmid type.

A plasmid stability assay revealed that the average frequency of HLGR loss in E250 was 2.54 × 10^−4^, similar to that observed in E37 (13). PCR and sequencing of non-HLGR E250 variants indicated that resistance loss resulted from homologous recombination between the intact and type III IS*1216V*-inserted *aac(6′)-Ie-aph(2″)-Ia*. No variants lacking the entire megaplasmid were detected.

### Distribution and genotypic analysis of *E. faecium* from 2011 to 2020

Given that the RepA_N-type megaplasmid enhanced the carbohydrate utilization repertoire in ST17 and ST78 *E. faecium*, its distribution was examined among 465 *E. faecium* isolates recovered from blood cultures between 2011 and 2020. As summarized in Table 1, *repA_N* was highly prevalent (overall 90.8%), with a range of 85.7%–96.2% across the study period. The sucrose utilization gene cluster, represented by *scrB* (sucrose-6-phosphate hydrolase), was detected in 85.5% of isolates. Among 183 *scrB*-positive isolates tested, all fermented sucrose, confirming functional expression.

**TABLE 1:** Temporal trends of RepA_N megaplasmid markers and antibiotic resistances in *E. faecium*, 2011 to 2020^*a*^

Year	No. of isolates	No. of isolates (%)				
		*repA_N*	*scrB*	IS*1216V-*inserted *aac(6')-Ie-aph(2'')-Ia*	HLGR	VR
2011	26	25 (96.2)	24 (92.3)	22 (84.6)	15 (57.7)	5 (19.2)
2012	42	38 (90.5)	36 (85.7)	31 (73.8)	25 (59.5)	3 (7.1)
2013	52	46 (88.5)	48 (92.3)	38 (73.1)	33 (63.5)	2 (3.8)
2014	42	36 (85.7)	36 (85.7)	33 (78.6)	22 (52.4)	1 (2.4)
2015	36	31 (86.1)	26 (72.2)	19 (52.8)	19 (52.8)	15 (41.7)
2016	49	42 (85.7)	35 (71.4)	35 (71.4)	24 (49.0)	16 (32.7)
2017	24	23 (95.8)	22 (91.7)	21 (87.5)	16 (66.7)	7 (29.2)
2018	59	56 (94.9)	49 (83.1)	39 (66.1)	26 (44.1)	29 (49.2)
2019	74	70 (94.6)	64 (86.5)	41 (55.4)	34 (45.9)	42 (56.8)
2020	61	55 (90.2)	59 (96.7)	34 (55.7)	28 (45.9)	39 (63.9)
Total	465	422 (90.8)	399 (85.8)	313 (67.3)	242 (52.0)	159 (34.2)

^*a*^ HLGR, high-level gentamicin resistance; VR, vancomycin resistance.

In contrast, the IS*1216V*-inserted *aac(6′)-Ie-aph(2″)-Ia* gene was found in 67.3% of isolates and showed a decreasing trend without reaching statistical significance (*P* = 0.087), consistent with a decline in the HLGR phenotype (*P* = 0.063). Meanwhile, the proportion of VREfm increased significantly during the same period (*P* = 0.001).

**Fig 4:**
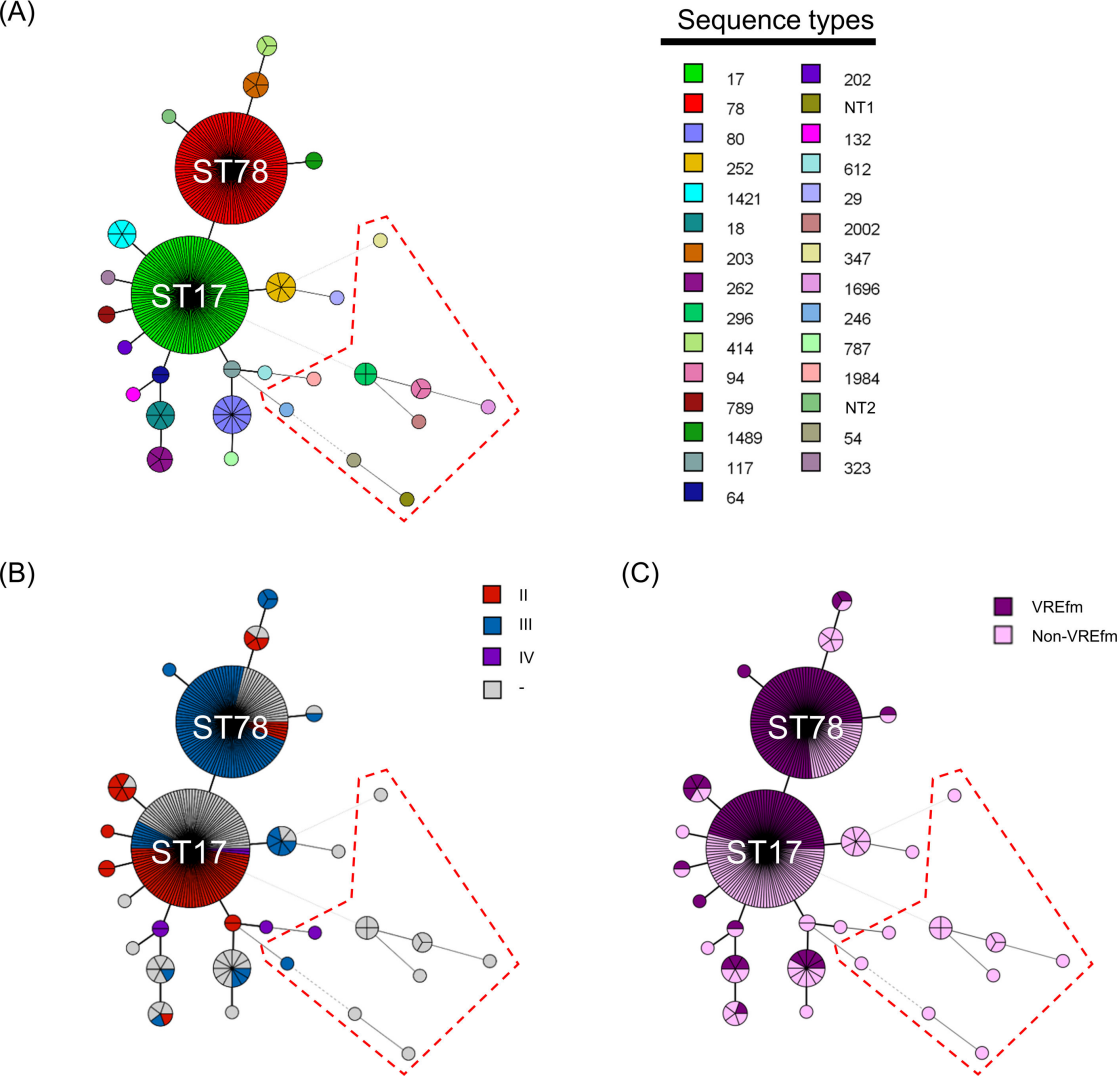
Minimum spanning tree of 298 *E. faecium* isolates constructed based on seven MLST loci. Each circle represents an ST, and the number of isolates is indicated by subdivision of the circle and is proportional to the circle size. Colors indicate (**A**) different STs, (**B**) presence and IS*1216V* insertion types, and (**C**) vancomycin resistance status (VREfm vs non-VREfm), as shown in the figure. The red dashed polygon highlights non-CC17 lineages. NT, newly identified ST.

PFGE analysis classified the 465 *E. faecium* isolates into 84 pulsotypes and 82 singletons (Fig. S2). Five pulsotypes (25, 34, 49, 60, and 64) contained more than 10 isolates (*n* = 14–49), accounting for 29.2% of the total collection. MLST was performed for 298 isolates, revealing 29 STs (Fig. 4A). ST17 and ST78, both members of the hospital-adapted CC17 lineage, were predominant and corresponded to the major pulsotypes (46 and 49 for ST17; 25 and 34 for ST78). Most ST78 isolates (78.5%, 84/107) carried the type III IS*1216V* insertion in *aac(6′)-Ie-aph(2″)-Ia*, whereas most ST17 isolates (52.5%, 62/118) carried the type II insertion (Fig. 4B).

Among the 465 *E. faecium* isolates, 159 were VREfm. ST78 (*n* = 82) and ST17 (*n* = 54) together accounted for the majority of VREfm isolates (Fig. 4C). The rapid expansion of ST78 *vanA*-positive VREfm since 2015 (Table S4) drove the emergence of the type III IS*1216V*-inserted *aac(6′)-Ie-aph(2″)-Ia*. In contrast, ST17, which predominantly carried the type II insertion, remained relatively stable owing to the persistence of its VSE members (pulsotypes 46 and 49) throughout 2011–2020, although an increase in ST17 VREfm was observed after 2018 (Table S4).

Notably, 14 isolates belonged to non-CC17 lineages, comprising nine STs (ST54, ST94, ST246, ST296, ST347, ST1696, ST1984, ST2002, and NT1) (Fig. 4). Among these, four STs (ST54, ST94, ST296, and ST1696; *n* = 9) belonged to CC94, corresponding to *E. lactis* in MLST databases. Moreover, although the ST1696 isolate and two other non-CC17 isolates (ST246 and ST1984) were positive for *repA_N*, only ST1696 did not carry the *aac(6′)-Ie-aph(2″)-Ia* and *scrB* genes.

## DISCUSSION

In this study, we identified a RepA_N-type megaplasmid enriched in the CC17 lineage over a decade in Taiwan (Table 1). This plasmid carried the gentamicin resistance gene *aac(6′)-Ie-aph(2″)-Ia* and multiple carbohydrate utilization gene clusters (Fig. 1 and 3). Consistent with our earlier findings (15), the presence of *aac(6′)-Ie-aph(2″)-Ia* and the rates of HLGR have declined among *E. faecium* isolates. In contrast, the presence of *repA_N* and the sucrose utilization gene cluster remained stable over the 10 years (Table 1). This divergence suggests that, while gentamicin resistance determinants may be lost in the absence of antibiotic pressure, traits associated with carbohydrate metabolism may impose less fitness cost and are more likely to be retained. These findings suggest a potential role of metabolic adaptation in the long-term success of clinically relevant *E. faecium* lineages in Taiwan.

Many *E. faecium* strains metabolize carbohydrates through PTS-mediated pathways (13). Maintaining metabolic versatility appears to be an essential strategy that enables *E. faecium* to persist within the host and potentially contribute to endogenous infections. In this study, the RepA_N-type megaplasmid identified in both ST17 and ST78 sublineages carried multiple gene clusters involved in PTS-mediated carbohydrate transport and utilization. Notably, the ability to utilize sucrose conferred by the RepA_N-type megaplasmid was likely important for the growth of ST17 *E. faecium* isolate E37 (Fig. 2). In *Streptococcus pneumoniae*, sucrose metabolism promotes nasopharyngeal colonization and lung infection in mouse models (16), while in *Enterococcus faecalis*, sucrose enhances biofilm formation and induces virulence-associated gene expression (17). Moreover, deletion of the universal PTS components PtsHI in *E. faecium* impairs biofilm formation and reduces competitive fitness (13). Collectively, these findings imply that acquisition of the RepA_N-type megaplasmid may facilitate adaptation to fluctuations in host-derived carbohydrate availability, thereby contributing to the persistence of *E. faecium* in clinical environments.

Our previous study revealed a marked reduction in gentamicin consumption at Kaohsiung Medical University Hospital (from 12.5 to 5.0 DDDs per 1,000 patients per day during 2011–2019) (15). In parallel, the current study demonstrated a decline in the proportion of isolates exhibiting the HLGR phenotype, consistent with the decreased prevalence of the IS*1216V*-inserted *aac(6′)-Ie-aph(2″)-Ia* gene (Table 1). In the stability assay, culturing the ST17 isolate E37 in antibiotic-free medium yielded variants that either lost the *aac(6′)-Ie-aph(2″)-Ia* gene or cured the entire plasmid (Fig. 1). Although plasmid loss observed *in vitro* may not fully reflect *in vivo* dynamics, these findings, together with the observed temporal trends, suggest a possible association between reduced selective pressure and decreased gentamicin resistance in *E. faecium*. However, a causal relationship cannot be established, and other confounding factors may also contribute.

ISs are well recognized for facilitating genetic rearrangements and niche adaptation in hospital-associated *E. faecium* (12, 18, 19). In this study, multiple IS elements were identified within the RepA_N-type megaplasmids of the two major sublineages, ST17 and ST78, consistent with the genomic characteristics typical of clinical *E. faecium*. The structural heterogeneity observed in the plasmids from isolates E37-21, E198, and E250 likely reflected IS-mediated rearrangements, exemplified by three distinct IS insertions in the RepA_N-type megaplasmid of E198 and E250 (Table S2). Such IS-mediated plasticity may contribute to the evolutionary diversification of specific clonal lineages.

To further understand the population dynamics associated with these plasmid variants, we analyzed the genetic structure of *E. faecium* isolates collected over 10 years. All *E. faecium* isolates collected in this study were recovered from blood cultures. Among the 298 isolates subjected to MLST, 95.3% (284/298) belonged to the CC17 lineage, consistent with the well-established association of CC17 (also referred to as clade A1) with hospital adaptation (12, 18, 20, 21). ST17 and ST78 were the predominant sublineages, typically harboring type II and type III IS*1216V*-inserted *aac(6′)-Ie-aph(2″)-Ia* genes, respectively (Fig. 4). ST78 emerged predominantly after 2015, coinciding with the rise of VREfm, whereas ST17 was consistently detected throughout the 10-year surveillance period and reemerged as one of the major VREfm sublineages after 2018. Similar population shifts of VREfm have been reported in Taiwan (22–28). The alternating predominance of ST17 and ST78 lineages may reflect fluctuating selection pressures and ecological niches within clinical environments.

As for non-CC17 isolates, 64.3% (9/14) were assigned to CC94 (ST94, ST296, ST1696, and ST2002), which was previously designated *E. faecium* clade B and reclassified as *E. lactis* in 2021 (29). Although MLST can provide some indication, it does not reliably distinguish *E. lactis* from *E. faecium*, as the scheme was originally developed for *E. faecium*. Due to their close phylogenetic relatedness, routine clinical identification methods often fail to differentiate these two species (29, 30). Given that the primary aim of this study was to investigate plasmid carriage and genetic context, and that these CC94 isolates represent only a minority of the data set, we retained them in the analysis as part of the non-CC17 lineages to reflect real-world clinical identification conditions.

This study has several limitations. First, only isolates recovered from blood cultures were included, and differences in megaplasmid carriage between infection sources cannot be fully excluded. However, our previous study (15), which included ST17 and ST78 isolates from urine, demonstrated similar carriage patterns of IS*1216V*-inserted *aac(6′)-Ie-aph(2″)-Ia*. Second, the current data set does not allow a definitive distinction between hospital- and community-associated isolates. Third, while carbohydrate utilization is known to facilitate intestinal colonization, its direct contribution to adaptation in clinical environments remains to be fully elucidated. Finally, the plasmid stability assay was conducted under *in vitro* conditions, which may not fully reflect the complex selective pressures present *in vivo*.

In summary, our findings underscore the potential role of the RepA_N-type megaplasmid in shaping the success of relevant *E. faecium* lineages in Taiwan. The linkage between antimicrobial resistance and metabolic versatility, particularly in carbohydrate utilization, may represent a key adaptive mechanism driving the persistence of CC17 lineages. The long-term maintenance of this megaplasmid suggests a favorable balance between its fitness cost and adaptive benefits under clinical selection pressures, such as antibiotic exposure and nutrient limitation.

## MATERIALS AND METHODS

### Bacterial strains

A total of 481 *E. faecium* isolates were recovered from blood cultures at Kaohsiung Medical University Hospital between 2011 and 2020 and at Kaohsiung Municipal Ta-Tung Hospital between 2017 and 2020, with only one isolate per patient included. Species identification was confirmed by enterococcal multiplex PCR or 16S rRNA gene sequencing (31). Finally, a total of 465 *E. faecium* isolates were identified. Susceptibility testing of gentamicin and vancomycin for *E. faecium* was performed by agar dilution method according to CLSI guidelines, and *E. faecalis* ATCC 29212 was used as the quality control strain (32). Detection of vancomycin-resistant genes was performed as previously described (33, 34). E37 with HLGR phenotype and its variants with non-HLGR phenotypes were obtained from our previous work (15).

### S1 nuclease-digested PFGE

The preparation of agarose-embedded bacterial DNA and the S1 nuclease-digested PFGE analysis were performed as previously described (15, 35). In brief, the agarose-embedded bacterial DNA was cut by 10 U of *Aspergillus oryzae* S1 nuclease (Invitrogen) at 37°C for 45 min. The reaction was stopped with ES buffer. Each band was considered a unit length of linear plasmid after separation by CHEF-DRIII apparatus in 1.2% (wt/v) agarose gels with a pulse angle of 120° and the pulse times of 45 s for 14 h and 25 s for 6 h at 200 V.

### Southern blot

The linear plasmids in the gel, separated by S1-PFGE, were transferred to a Hybond-N+ nylon membrane (GE Healthcare) by vacuum blotting and then fixed under UV light. The DNA probe specific for *aac(6′)-Ie-aph(2″)-Ia* gene was labeled with digoxigenin (DIG) by PCR. The membrane was hybridized with the probe in DIG Easy Hyb buffer (Roche Diagnostics GmbH). Detection of hybridization was performed using alkaline phosphatase-conjugated anti-DIG antibody (Roche Diagnostics GmbH) and the substrate CSPD (Roche Diagnostics GmbH) according to the manufacturer’s instructions.

### Next-generation sequencing

Whole-genome sequencing of the ST17 E37 variants 21 and 24 (E37-21 and E37-24), ST78 *E. faecium* E198 and E250 was conducted using a PacBio Sequel platform (Pacific Bioscience, Menlo, CA). A 10–20 kb insert library was prepared using the Express Template Preparation Kit 2.0, and sequencing was performed using continuous long-read (CLR) sequencing mode. HiFi reads generated from CLR were conducted using SMRT Analysis version 10.2 (Pacific Biosciences, Menlo Park, CA). *De novo* assembly was performed using Flye v.2.9 (36). The annotation was determined by Prokka (37) and NCBI BLAST. The *rep* genes (replication initiator genes) of plasmid types were predicted by PlasmidFinder (https://cge.food.dtu.dk/services/PlasmidFinder/) (38).

### Carbohydrate fermentation testing

BBL phenol red broth (Becton Dickinson), with or without 1% (wt/vol) individual carbohydrates, including fructose, galactose, glucose, lactose, mannitol, melibiose, rhamnose, and sucrose, was prepared according to the manufacturer’s instructions (39). Selected *E. faecium* isolates were inoculated into 200 μL of phenol red broth in 96-well plates. After incubation at 37°C for 24 h, visible bacterial growth and a color change to yellow were observed, indicating positive fermentation of the corresponding carbohydrate. Each experiment was performed in duplicate. For strain E37 and its variants (E37-21, E37-24, E37-26, and E37-27), growth curves were monitored using an XS2 ELISA reader (Biotex, Inc.).

### Detection of the RepA_N megaplasmid

Detection of the RepA_N megaplasmid was performed by PCR using five primer sets (Table S5). Two sets targeting the replication initiator gene *repA_N* and the sucrose-6-phosphate hydrolase gene *scrB*, respectively, and three sets targeting the IS*1216V*-inserted *aac(6′)-Ie-aph(2″)-Ia* gene and its insertion types, as previously described (15, 40). PCR amplification was carried out using rTaq (Takara) in a 25 μL reaction volume. The cycling conditions were as follows: initial denaturation at 94°C for 5 min, followed by 30 cycles of denaturation at 94°C for 30 s, annealing at 50°C for 30 s, and extension at 72°C for 30–90 s.

### Multilocus sequence typing

Multilocus sequence typing (MLST) of the *E. faecium* was performed as previously described (41). The STs and CCs were determined by submission of the DNA sequences of the seven loci to the MLST database (https://pubmlst.org/organisms/enterococcus-faecium).

### Stability assay of gentamicin resistance

The ST78 HLGR *E. faecium* strain E250 with a type III IS*1216V*-inserted *aac(6*′)*-Ie-aph(2*″)*-Ia* and an intact *aac(6*′)*-Ie-aph(2″)-Ia* was chosen to evaluate the probability (*P*) of loss of HLGR phenotype by the stability assay, as previously described (15, 42). In brief, the number of generations (*n*) was determined by counting the population at 0 h and 5.5 h during the logarithmic growth. The frequency of HLGR loss (*x*) was defined as the number of colonies with non-HLGR/total number of colonies by picking around 1,000 colonies growing at 5.5 h. Finally, the probability (*P*) of loss of HLGR was determined using the equation: *P* = 1-$\sqrt[{n}]{1-x}$. The stability assay was carried out in triplicate. The surrounding genetic organization of the *aac(6*′)*-Ie-aph(2″)-Ia* was determined using PCR mapping as described above.

### Statistics

Statistical analyses were performed using IBM SPSS Statistics for Windows, Version 20.0 (Armonk, NY, USA). Linear regression analysis was used to assess temporal trends from 2011 to 2020. A *P* value of < 0.05 was considered statistically significant.

## Data Availability

The *aac(6′)-Ie-aph(2″)*-Ia-carrying plasmids of E37-21, E198, and E250 determined in this study were submitted to GenBank under accession numbers OQ883946 to OQ883948.

## SUPPLEMENTAL MATERIAL

Figure S1 spectrum.03793-25-s0001.pdf Sequence comparison between plasmids pE37-21 and pE250.

Figure S2 spectrum.03793-25-s0002.pdf PFGE analysis of SmaI-digested DNA of 465 *E. faecium* isolates in Taiwan, 2011 to 2020.

Supplemental tables spectrum.03793-25-s0003.pdf Tables S1 to S5.

ASM does not own the copyrights to Supplemental Material that may be linked to, or accessed through, an article. The authors have granted ASM a non-exclusive, world-wide license to publish the Supplemental Material files. Please contact the corresponding author directly for reuse.
