# Supplementary material for: Widespread dissemination of the RepA_N-type megaplasmid conferring a carbohydrate utilization advantage in clonal complex 17 Enterococcus faecium in Taiwan
Source: Microbiol Spectr. 2026 Jul 14;14(8):e03793-25. doi: 10.1128/spectrum.03793-25 (PMC13436390; doi:10.1128/spectrum.03793-25)
Supplement: Supplementary file 1 [file spectrum.03793-25-s0001.pdf]

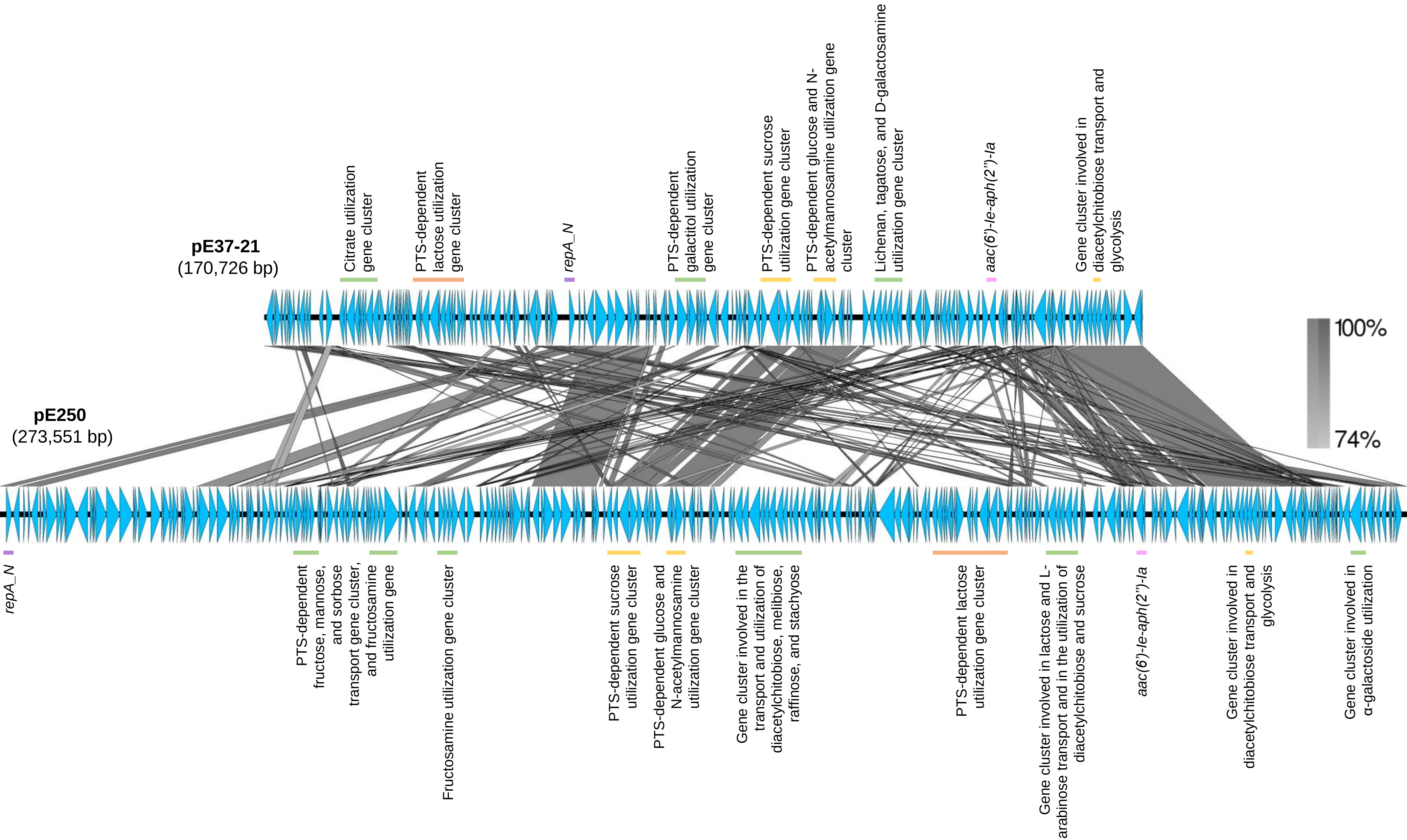

Fig. S1. Sequence comparison between plasmids pE37-21 and pE250. Blue arrows indicate open reading frames or truncated genes. Grey shading represents homologous regions, with similarity indicated by the scale. Green lines indicate gene clusters unique to the corresponding plasmid. Orange lines indicate gene clusters present in both plasmids but highly divergent at the nucleotide level. Yellow lines indicate gene clusters conserved in both plasmids. Purple lines indicate the *repA_N* gene. Pink lines indicate the *aac(6')-Ie-aph(2")-Ia* gene.
