# Supplementary material for: Widespread dissemination of the RepA_N-type megaplasmid conferring a carbohydrate utilization advantage in clonal complex 17 Enterococcus faecium in Taiwan
Source: Microbiol Spectr. 2026 Jul 14;14(8):e03793-25. doi: 10.1128/spectrum.03793-25 (PMC13436390; doi:10.1128/spectrum.03793-25)
Supplement: Supplementary file 2 [file spectrum.03793-25-s0002.pdf]

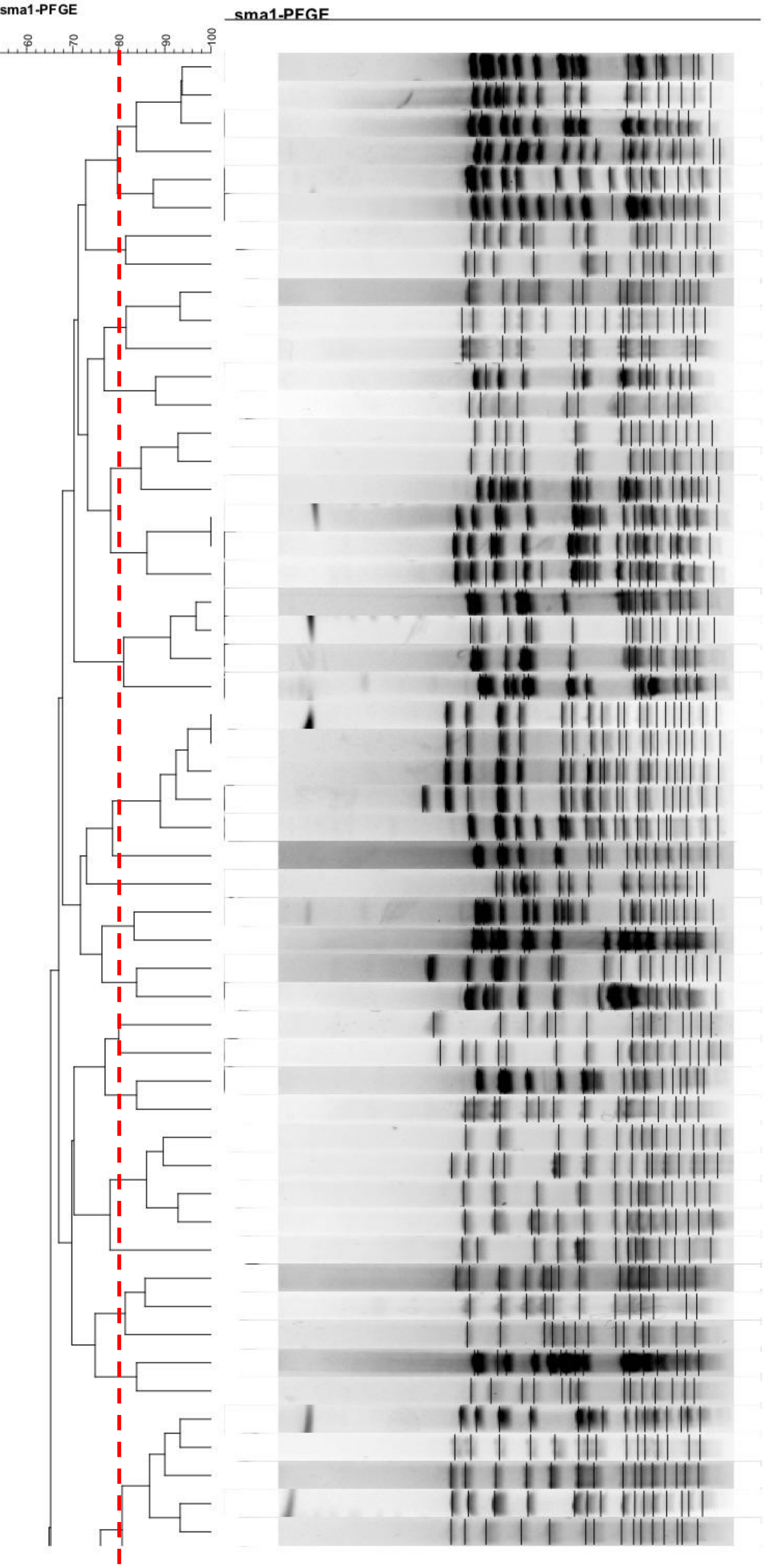

NO.	Year	Van	Gen	*repA_N*	*scrB*	Insert	ST	
E35	2012	S	R	-	+	II	262	Pulsotype 1
E140	2014	S	R	+	+	III	262	
E315	2018	S	S	+	+	-		
E52	2012	R	R	-	+	-	262	
E88	2013	S	S	+	+	-		Pulsotype 2
E160	2014	S	S	+	-	III	262	
E436	2020	S	R	+	+	II	789	Pulsotype 3
E477	2020	S	R	+	+	-		
E352	2019	S	R	+	+	III	80	Pulsotype 4
E407	2019	R	S	+	+	III	78	
E429	2020	S	S	-	-	-	18	
E252	2015	R	S	-	-	III	18	Pulsotype 5
E202	2016	S	R	+	+	-		
E459	2020	R	S	+	+	-	80	Pulsotype 6
E443	2020	R	S	+	+	-	80	
E181	2015	S	S	+	+	-	17	
E200	2016	R	S	+	+	II	1421	Pulsotype 7
E253	2015	R	S	+	+	II	1421	
E255	2016	R	S	+	+	II	1421	
E212	2016	S	R	+	-	III	80	Pulsotype 8
E289	2018	S	R	+	+	-		
E228	2016	S	R	+	-	III		
E177	2015	S	R	+	+	II	117	
E59	2012	S	S	+	+	II	203	Pulsotype 9
E112	2013	S	S	+	+	II	203	
E111	2013	S	S	+	+	II		
E90	2013	S	S	+	+	II		
E89	2013	S	S	+	+	-		
E23	2011	R	R	+	+	II	17	
E192	2015	S	R	+	-	-		
E102	2013	S	R	+	+	II	262	Pulsotype 10
E342	2018	S	S	+	-	-		
E01	2011	S	S	-	+	II	203	Pulsotype 11
E288	2018	R	S	+	+	III	78	
E440	2020	R	R	+	+	III	17	
E447	2020	S	R	+	+	-	17	
E32	2012	S	R	+	+	II	17	Pulsotype 12
E428	2020	S	R	+	-	-		
E444	2020	R	S	+	+	II	17	Pulsotype 13
E445	2020	S	S	+	+	-	78	
E438	2020	R	R	+	+	II	17	
E476	2020	R	R	+	+	II	17	
E437	2020	R	S	+	+	II	17	
E374	2019	R	R	+	+	III	17	Pulsotype 14
E426	2020	R	R	+	+	III	17	
E142	2014	S	S	-	-	-	29	
E334	2018	S	R	+	+	-		Pulsotype 15
E109	2013	S	S	-	+	-	17	
E176	2015	R	S	+	+	-	1421	Pulsotype 16
E396	2019	S	R	-	+	-	17	
E358	2019	R	R	+	+	-	17	
E259	2017	S	R	+	+	II	1421	
E122	2013	R	R	+	+	-	17	

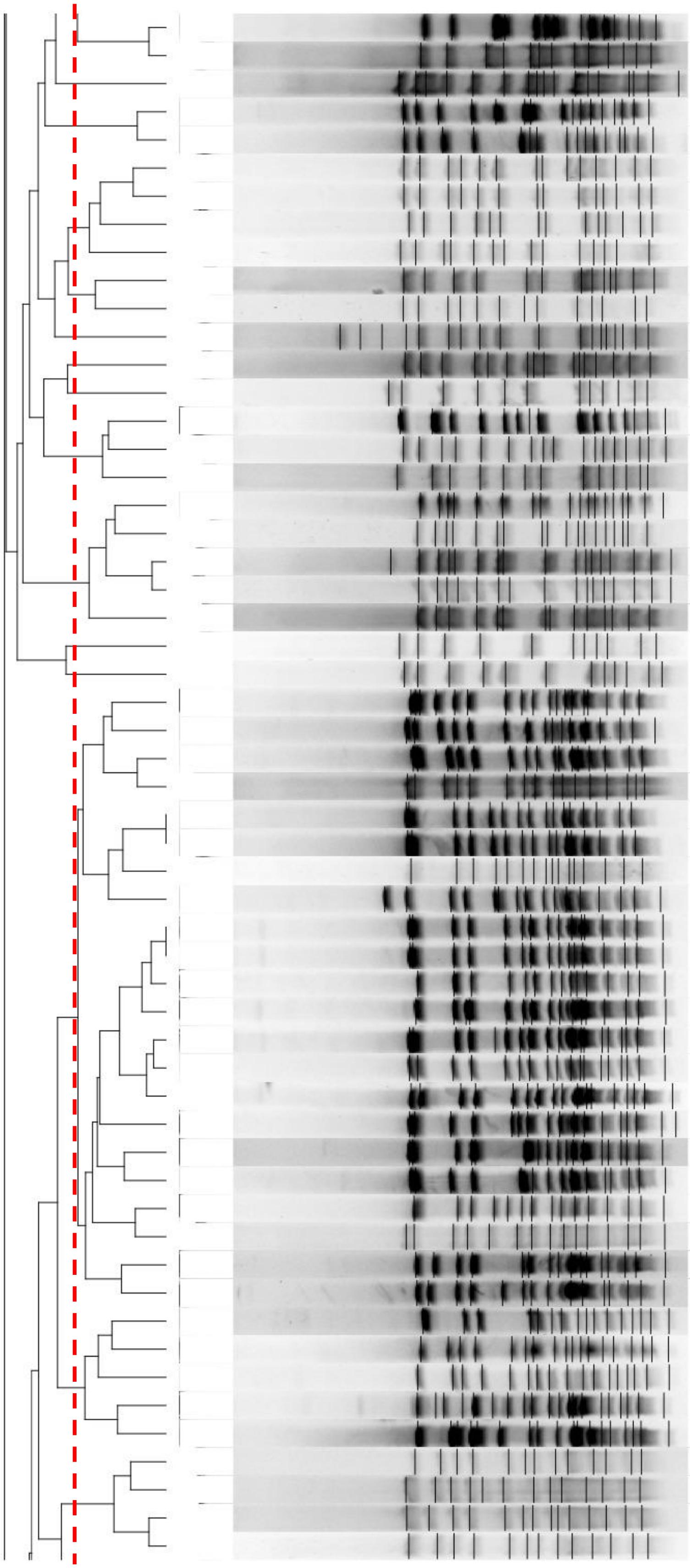

NO.	Year	Van	Gen	*repA_N*	*scrB*	Insert	ST	
E163	2014	S	R	+	+	II	17	Pulsotype 17
E372	2019	S	S	+	+	II	17	
E367	2019	R	R	+	+	IV	17	
E305	2018	R	R	+	+	III	17	Pulsotype 18
E311	2018	S	S	+	+	II	1421	
E399	2019	R	R	+	+	-	17	Pulsotype 19
E406	2019	S	R	+	+	-	17	
E455	2020	R	R	+	+	-	17	
E417	2019	S	R	+	-	-	17	
E344	2019	S	S	-	+	II	132	Pulsotype 20
E402	2019	R	R	+	+	-	17	
E353	2019	R	R	+	+	-	17	
E381	2019	R	R	+	+	-	17	
E401	2019	S	R	+	+	II	17	
E238	2016	S	R	+	+	III	17	Pulsotype 21
E478	2020	R	S	-	+	-	17	
E346	2019	S	S	+	+	III	17	
E298	2018	S	R	+	+	-		Pulsotype 22
E430	2020	S	R	+	+	-		
E368	2019	S	R	+	+	-		
E385	2019	S	R	+	-	-		
E380	2019	S	R	+	+	-		
E410	2019	R	S	+	+	II	17	
E435	2020	R	R	+	+	-	17	
E286	2018	R	S	+	+	-	78	Pulsotype 23
E303	2018	R	R	+	+	-	78	
E296	2018	R	S	+	+	III	78	
E364	2019	R	S	+	+	III	78	
E306	2018	R	S	+	+	III	78	Pulsotype 24
E312	2018	R	S	+	+	III	78	
E319	2018	R	S	+	+	III	78	
E270	2017	R	R	+	+	III	78	
E248	2015	R	R	+	+	III	78	Pulsotype 25
E249	2015	R	R	+	+	III	78	
E251	2015	R	R	+	+	III	78	
E240	2016	R	S	+	+	-	78	
E250	2015	R	R	+	+	III	78	
E268	2017	S	S	+	+	II	78	
E257	2017	S	R	+	+	III	78	
E245	2016	R	S	+	+	III	78	
E217	2016	S	S	+	+	II	17	
E297	2018	S	S	+	+	III	78	
E285	2017	R	R	+	+	III	17	
E388	2019	R	S	+	+	III	78	
E218	2016	R	S	+	+	III	78	
E221	2016	R	R	+	+	IV	17	
E92	2013	S	S	+	+	-	78	Pulsotype 26
E254	2016	R	R	+	+	II	78	
E264	2017	R	R	+	+	III	78	
E246	2015	R	R	+	-	III	78	
E316	2018	R	S	+	+	III	78	
E389	2019	R	S	+	+	III	78	Pulsotype 27
E392	2019	R	R	+	+	III	78	
E393	2019	S	S	+	+	III	78	
E413	2019	R	R	+	+	III	78	

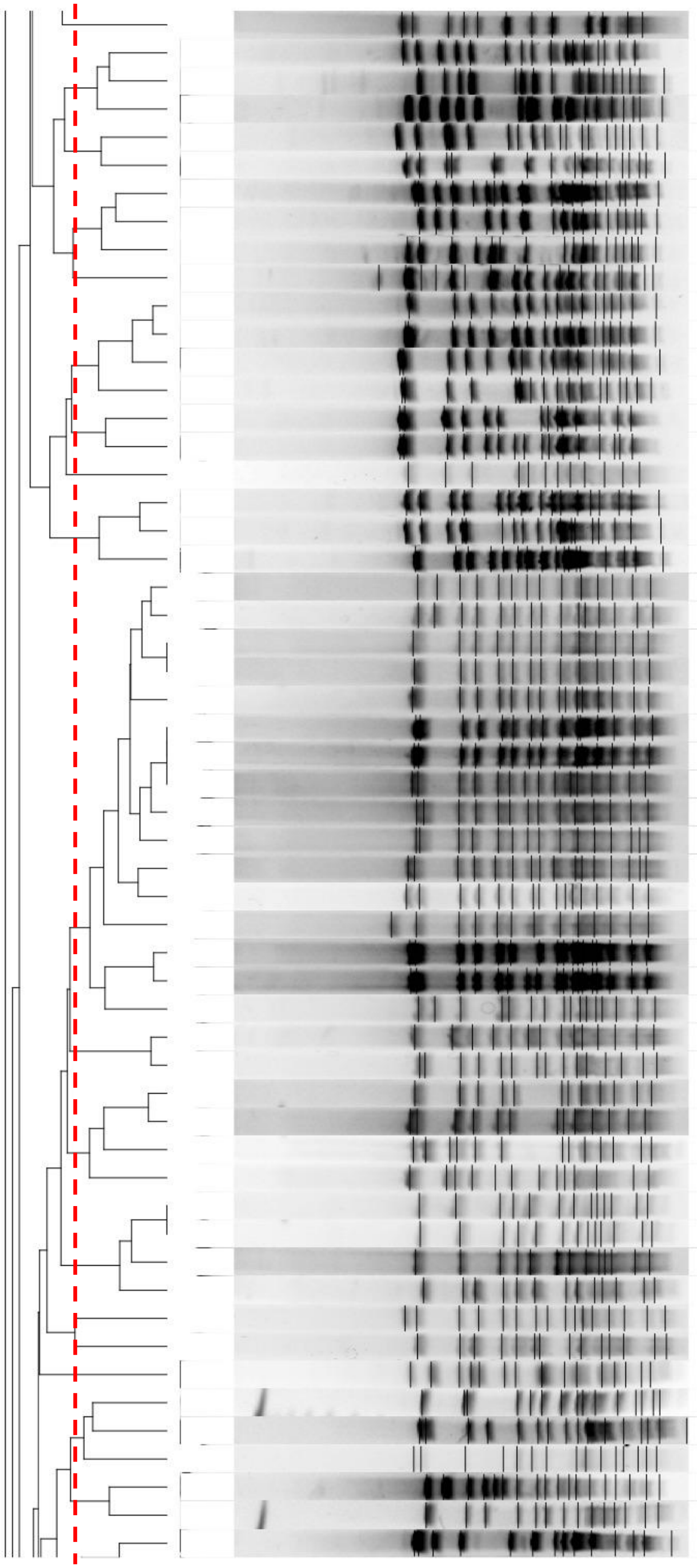

NO.	Year	Van	Gen	*repA_N*	*scrB*	Insert	ST	
E333	2018	S	S	+	-	-		
E302	2018	R	R	+	-	III	17	Pulsotype 28
E314	2018	R	R	+	+	III	78	
E36	2012	S	R	+	+	-	78	
E127	2014	S	S	+	+	II	203	Pulsotype 29
E284	2017	R	S	+	+	III	17	
E299	2018	R	R	+	+	-	17	Pulsotype 30
E301	2018	R	R	+	+	II	17	
E308	2018	R	S	+	+	III	17	
E309	2018	R	S	+	+	III	78	
E304	2018	R	S	+	+	III	78	Pulsotype 31
E307	2018	R	S	+	+	III	78	
E279	2017	R	S	+	+	III	78	
E269	2017	S	R	+	+	III	78	
E277	2017	R	S	+	+	III	78	Pulsotype 32
E278	2017	R	R	+	+	III	78	
E321	2018	S	R	+	+	III	78	
E271	2017	R	S	+	+	III	78	Pulsotype 33
E274	2017	R	S	-	+	-	78	
E247	2015	R	R	+	+	III	78	
E351	2019	R	S	+	+	III	78	Pulsotype 34
E468	2020	R	S	+	+	III	78	
E348	2019	R	S	+	+	III	78	
E359	2019	R	S	+	+	III	78	
E343	2018	R	R	+	+	III	78	
E332	2018	R	S	+	+	III	78	
E335	2018	R	S	+	+	III	78	
E379	2019	R	R	+	+	III	78	
E376	2019	R	S	+	+	-	78	
E355	2019	R	S	+	+	III	78	
E354	2019	R	S	+	+	III	78	
E398	2019	R	S	+	+	III	78	
E347	2019	R	S	+	-	III	78	
E338	2018	R	R	+	+	III	78	
E339	2018	R	S	+	+	III	78	
E471	2020	R	S	+	+	-	78	
E472	2020	R	R	+	+	-	78	Pulsotype 35
E480	2020	R	S	+	+	III	78	
E357	2019	R	S	+	+	III	78	Pulsotype 36
E365	2019	R	S	+	+	III	78	
E434	2020	R	S	+	+	III	1489	
E431	2020	R	S	+	+	III	78	
E405	2019	R	R	+	+	III	78	Pulsotype 37
E408	2019	S	R	+	+	III	78	
E361	2012	S	S	+	+	-	78	
E465	2020	R	R	+	+	III	78	
E439	2020	R	R	+	+	III	78	
E441	2020	R	R	+	+	III	78	
E144	2014	S	S	+	+	II	17	
E172	2015	R	S	+	+	-	78	Pulsotype 38
E204	2016	R	R	+	+	III	78	
E414	2019	R	R	+	+	III	80	
E81	2013	S	R	+	+	IV	64	Pulsotype 39
E201	2016	S	R	+	+	-		
E197	2016	R	R	+	+	III	78	Pulsotype 40

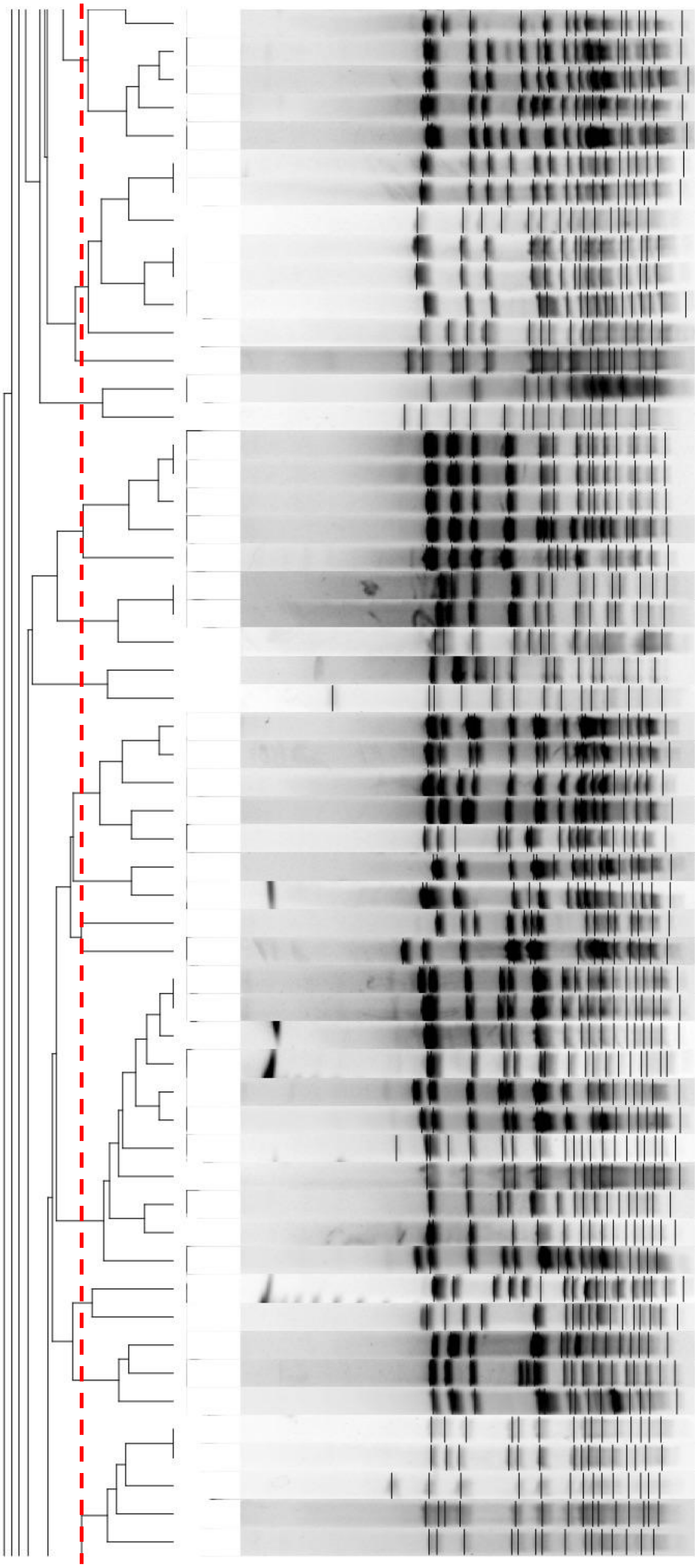

NO.	Year	Van	Gen	*repA_N*	*scrB*	Insert	ST	Pulsotype
E199	2016	R	R	+	+	III	78	Pulsotype 40
E178	2015	R	R	+	+	III	78	Pulsotype 41
E206	2016	S	S	+	+	III	78	
E198	2016	R	S	+	+	III	78	
E207	2016	R	R	+	+	III	78	
E292	2018	R	S	+	+	III	78	Pulsotype 42
E293	2018	R	S	+	+	III	78	
E328	2018	R	S	+	+	III	78	
E261	2017	S	S	+	+	III	78	
E262	2017	S	S	+	+	III	78	
E233	2016	R	R	+	+	III	78	
E475	2020	R	S	+	+	III	78	
E360	2019	R	S	+	+	-	17	
E167	2015	R	S	-	+	-	78	Pulsotype 43
E320	2018	R	R	+	+	-	17	
E75	2013	S	R	+	+	II	17	Pulsotype 44
E76	2013	S	R	+	+	II		
E74	2013	S	R	+	+	II		
E47	2012	S	R	+	+	II	17	
E78	2013	S	S	-	-	-	347	
E13	2011	S	S	+	+	-		Pulsotype 45
E14	2011	S	S	+	+	II	323	
E464	2020	S	S	+	+	-		
E136	2014	S	S	-	+	II	203	Pulsotype 46
E324	2018	S	S	-	-	-	2002	
E184	2015	S	R	+	+	-		Pulsotype 47
E223	2016	S	S	+	+	III	246	
E313	2018	S	S	+	+	-		
E48	2012	S	S	-	-	-	17	
E281	2017	S	R	+	+	II	17	
E210	2016	S	S	+	-	III	18	Pulsotype 48
E318	2018	S	R	+	+	-	17	
E186	2015	S	S	+	-	-		
E224	2016	S	R	+	+	-		
E106	2013	S	S	+	+	-	-	Pulsotype 49
E107	2013	S	R	+	+	III	252	
E86	2013	S	R	+	+	III		
E87	2013	S	R	+	+	III	252	
E97	2013	S	R	+	+	II		
E155	2014	S	S	+	+	-	252	
E116	2013	S	R	+	+	III		
E369	2019	R	S	+	+	-	17	
E125	2014	S	R	+	+	III	252	
E139	2014	S	S	+	-	-		
E164	2014	S	S	+	+	III		
E230	2016	S	S	+	+	-		Pulsotype 50
E287	2018	R	S	+	+	III	17	
E50	2012	S	R	+	+	II	17	Pulsotype 51
E94	2013	S	S	+	+	-		
E153	2014	S	S	+	+	-		
E421	2020	R	S	+	+	II	17	Pulsotype 52
E449	2020	S	S	+	+	-	17	
E397	2019	R	R	+	+	-	17	
E345	2019	S	R	+	+	-		
E165	2014	R	R	+	+	III	78	

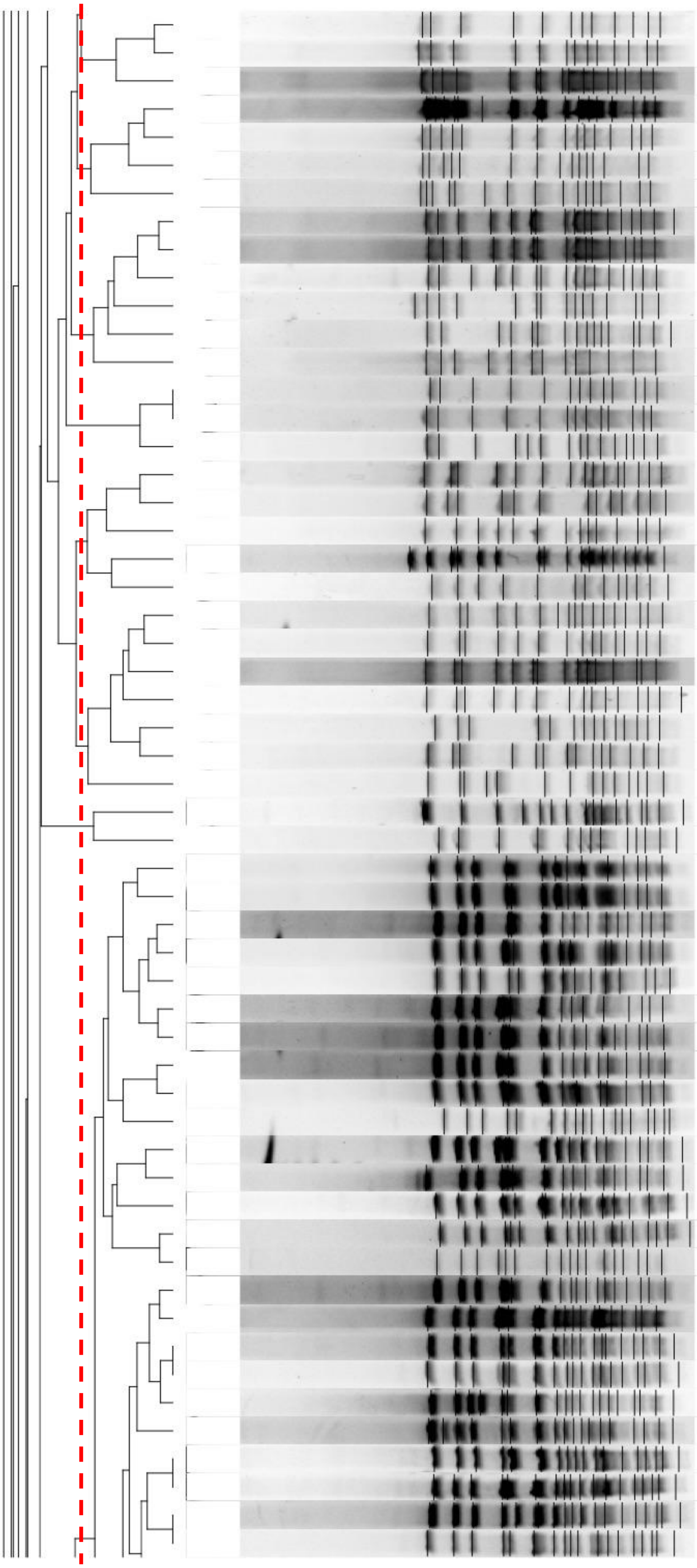

NO.	Year	Van	Gen	*repA_N*	*scrB*	Insert	ST	
E411	2019	R	R	+	+	-	17	Pulsotype 53
E432	2020	S	S	+	+	-	17	
E378	2019	S	R	+	+	-	17	
E340	2018	S	R	+	+	-	17	Pulsotype 54
E404	2019	S	R	+	+	-	17	
E470	2020	R	S	-	+	-	17	
E391	2019	S	R	+	+	-	17	
E363	2019	R	S	+	+	II	17	Pulsotype 55
E377	2019	R	S	+	+	II	17	
E462	2020	R	S	+	+	II	17	
E382	2019	S	R	+	+	-	17	
E395	2019	R	S	+	-	II	17	
E386	2019	R	S	+	+	II	17	
E169	2015	R	R	+	+	-	17	Pulsotype 56
E171	2015	R	R	+	+	-	17	
E457	2020	R	R	+	+	-	252	
E469	2020	R	S	+	+	II	17	Pulsotype 57
E479	2020	S	R	+	+	-		
E433	2020	R	R	+	+	II	17	
E220	2016	R	R	+	+	III	NT2	
E326	2018	S	R	+	+	IV	612	
E383	2019	R	S	+	+	II	17	Pulsotype 58
E460	2020	R	R	+	+	II	17	
E373	2019	R	R	+	+	II	17	
E420	2020	R	R	+	+	II	17	
E456	2020	R	S	+	+	-	80	
E457	2020	S	R	+	+	-	252	
E461	2020	R	R	+	+	II	17	
E235	2016	S	R	+	-	III	80	Pulsotype 59
E454	2020	R	S	+	+	II	17	
E157	2014	S	R	+	+	II		Pulsotype 60
E158	2014	S	R	+	+	II		
E15	2011	S	R	+	+	II		
E150	2014	S	R	+	+	III	252	
E145	2014	S	R	+	+	II		
E03	2011	S	R	+	+	II	17	
E21	2011	S	R	+	+	II	17	
E28	2012	S	R	+	+	II		
E148	2014	S	R	+	+	II		
E173	2015	S	S	+	+	II	17	
E30	2012	S	R	+	+	II		
E55	2012	S	R	+	+	II		
E232	2016	S	S	+	+	II	17	
E205	2016	S	R	+	+	II		
E226	2016	S	R	-	+	III	17	
E19	2011	S	S	+	+	II	17	
E341	2018	S	S	+	+	II		
E99	2013	S	R	+	+	II	17	
E263	2017	S	R	+	+	II	17	
E83	2013	S	S	+	+	-		
E100	2013	S	R	+	+	II		
E236	2016	S	R	+	+	II		
E237	2016	S	S	+	+	II		
E93	2013	S	R	+	+	II		
E266	2017	S	R	+	+	II		

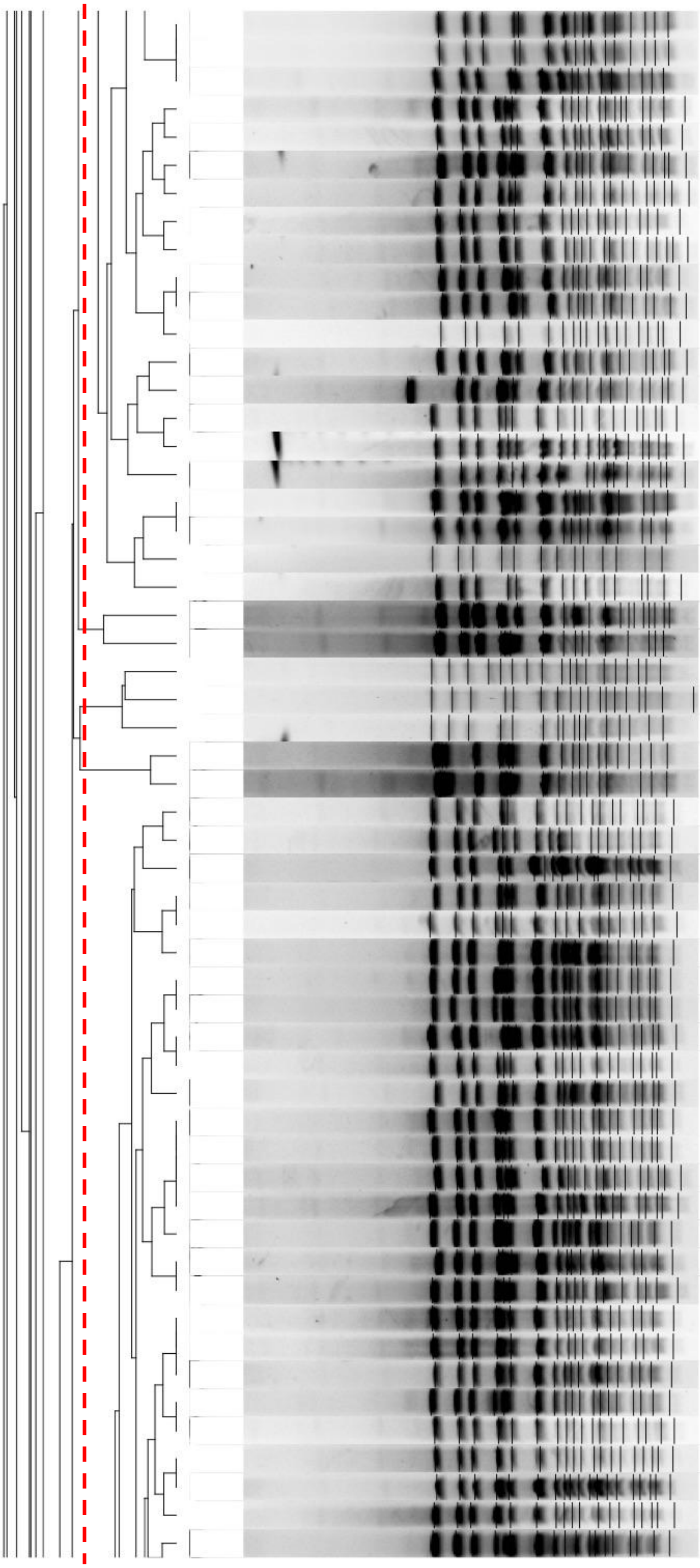

NO.	Year	Van	Gen	*repA_N*	*scrB*	Insert	ST	
E151	2014	S	R	+	+	II		
E152	2014	S	R	+	+	II	117	
E154	2014	S	R	+	+	II		
E60	2012	S	S	+	+	II		
E294	2018	S	R	+	+	II	17	
E57	2012	S	S	+	+	II		
E113	2013	S	R	+	+	II		
E84	2013	S	R	+	+	II		
E114	2013	S	R	+	+	II		
E53	2012	S	R	+	+	II	17	
E56	2012	S	R	+	+	II		Pulsotype 60
E239	2016	S	S	+	+	II		
E31	2012	S	R	+	+	II		
E108	2013	S	R	+	+	II		
E118	2013	S	R	+	+	II		
E258	2017	S	R	+	+	II		
E229	2016	S	S	+	+	II		
E147	2014	S	S	+	+	II		
E159	2014	S	S	+	+	II		
E356	2019	S	R	+	+	II	17	
E141	2014	S	S	-	-	-	17	
E38	2012	S	S	+	+	II		
E20	2012	S	S	+	+	II		Pulsotype 61
E85	2013	S	R	-	+	II	17	
E135	2014	S	R	+	+	II	17	Pulsotype 62
E65	2012	S	S	+	+			
E05	2011	S	R	+	+	II		
E17	2011	S	R	+	+	II	17	Pulsotype 63
E119	2013	S	R	+	+	II		
E124	2014	S	R	+	+	II		
E216	2016	S	R	+	-	-		
E49	2012	S	S	+	+	II		
E267	2017	S	R	+	+	II		
E42	2012	S	S	+	-	III		
E43	2012	S	R	+	+	II		
E44	2012	S	R	+	+	II		
E37	2012	S	R	+	+	II		
E131	2014	S	R	-	+	II	17	
E183	2015	S	S	+	+	II		Pulsotype 64
E69	2012	S	S	+	+	II		
E73	2013	S	R	+	+	II		
E95	2013	S	R	+	+	II		
E103	2013	S	S	+	+	II		
E41	2012	S	R	+	+	II		
E51	2012	S	R	+	+	II		
E101	2013	S	R	+	+	II		
E80	2013	S	R	+	+	II		
E82	2013	S	R	+	+	II	17	
E188	2015	S	R	+	+	II		
E71	2012	S	S	+	+	II		
E117	2013	S	R	+	+	II		
E132	2014	S	R	+	+	II		
E185	2015	S	S	+	+	II	17	
E133	2014	S	R	+	+	II		
E46	2012	S	R	+	+	II	17	

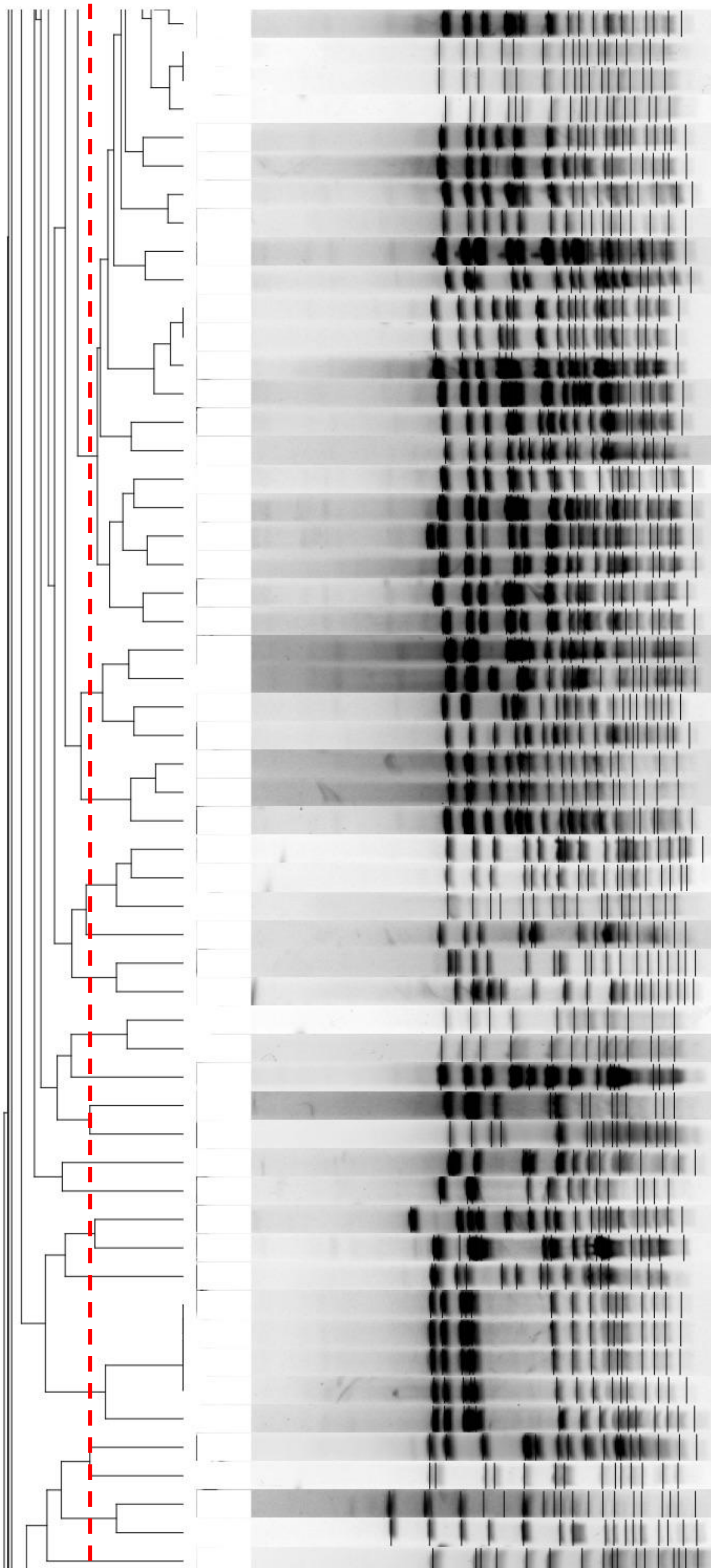

NO.	Year	Van	Gen	*repA_N*	*scrB*	Insert	ST	Pulsotype
E77	2013	S	R	+	+	II		Pulsotype 64
E323	2018	S	R	+	+	II		
E325	2018	S	S	+	+	II	17	
E337	2018	S	R	+	+	II		
E70	2012	S	S	+	+	II		
E79	2013	S	R	+	+	II		
E62	2012	S	R	+	+	II	17	
E126	2014	S	S	+	+	II		
E38	2012	S	S	+	+	II		
E179	2015	S	R	+	+	II		
E273	2017	S	R	+	+	II		
E275	2017	S	R	+	+	II	17	
E310	2018	S	R	+	+	II		
E40	2012	S	R	+	-	II		
E187	2015	S	R	+	+	II		
E213	2016	S	R	+	+	II	17	
E61	2012	S	R	+	+	II	17	
E96	2013	S	R	+	+	II		
E98	2013	S	S	+	+	II	17	
E104	2013	S	S	-	-	-	17	
E67	2012	S	R	+	+	II		
E105	2013	S	R	+	+	II		
E22	2011	R	R	+	+	IV		Pulsotype 65
E27	2011	R	R	+	+	IV		
E72	2013	R	R	+	+	IV	64	
E194	2015	S	R	+	+	II	17	
E07	2011	S	S	+	+	IV		Pulsotype 66
E08	2011	S	R	+	+	IV		
E33	2012	R	R	+	+	IV		
E231	2016	S	S	+	-	II	17	Pulsotype 67
E260	2017	S	S	+	+	II	17	
E384	2019	R	R	+	+	III	18	
E222	2016	R	R	+	+	III	78	
E91	2013	S	S	+	+	-	78	Pulsotype 68
E291	2018	S	R	+	-	IV		
E195	2015	S	R	+	+	II	17	Pulsotype 69
E162	2014	S	R	+	+	II	17	
E156	2014	S	S	+	+	II	17	
E09	2011	S	S	+	-	III		
E166	2014	S	S	+	+	-		
E54	2012	S	S	-	-	-	17	
E121	2013	S	S	+	+	II	78	
E68	2012	S	R	+	+	III	78	Pulsotype 70
E244	2011	R	R	+	+	II	414	
E280	2017	S	S	+	+	II	17	
E128	2014	S	R	+	+	III		Pulsotype 71
E129	2014	S	R	+	+	III		
E130	2014	S	R	+	+	III	78	
E137	2014	S	R	+	+	III		
E110	2013	S	R	+	+	III	78	
E161	2014	S	S	+	+	-		
E448	2020	R	S	+	+	-	17	
E12	2011	S	S	+	+	-		Pulsotype 72
E234	2016	S	S	-	-	-	94	
E446	2020	S	R	+	+	-		

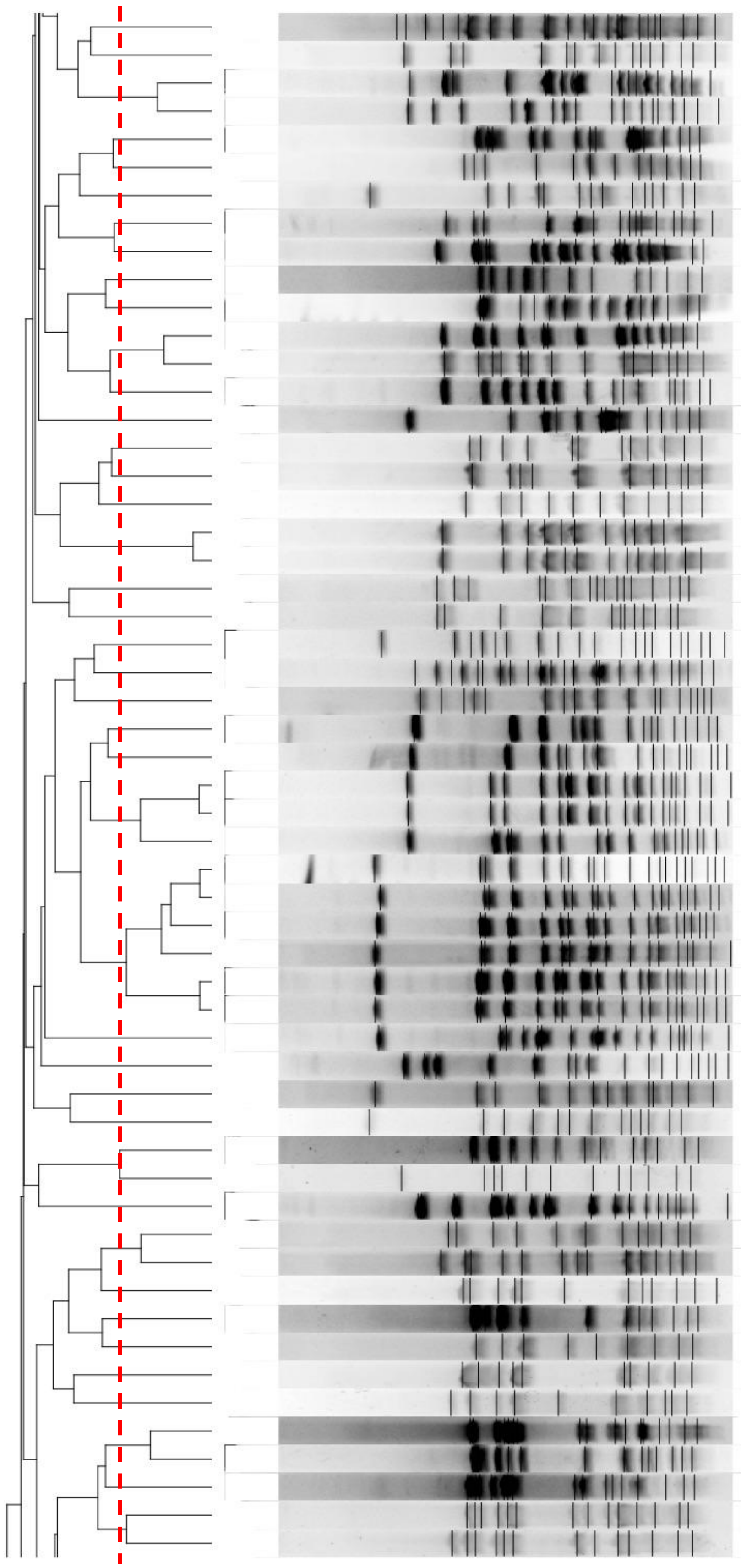

NO.	Year	Van	Gen	*repA_N*	*scrB*	Insert	ST	
E366	2019	S	S	-	+	-		
E425	2020	S	S	+	+	-		
E256	2016	R	S	+	+	II	78	Pulsotype 73
E276	2017	S	S	+	-	-	1696	
E146	2014	S	S	+	+	IV		
E170	2015	R	R	+	+	-	17	
E329	2018	S	S	+	-	-		
E225	2016	S	S	-	+	II	1489	
E272	2017	S	R	+	+	-	17	
E11	2011	S	R	+	+	-		
E143	2014	S	S	-	-	-	NT1	
E300	2018	S	R	+	+	III	17	Pulsotype 74
E467	2020	S	R	+	+	III	80	
E123	2013	S	S	+	+	-		
E331	2018	S	S	+	-	-		
E400	2019	S	S	+	-	-		
E473	2020	R	R	+	+	II	789	
E409	2019	S	S	+	+	-		
E466	2020	R	S	+	+	II	78	Pulsotype 75
E474	2020	R	S	+	+	II	78	
E387	2019	R	S	+	+	III	78	
E394	2019	R	S	+	+	II	78	
E120	2013	S	S	-	-	II	94	
E182	2015	S	S	-	-	-		
E370	2019	S	S	+	-	-		
E34	2012	S	S	+	+	-		
E138	2014	S	S	+	+	III	252	
E242	2016	S	S	+	-	-		Pulsotype 76
E243	2016	S	S	+	-	-		
E295	2018	S	S	-	+	III	94	
E115	2013	S	S	-	-	-	296	Pulsotype 77
E208	2016	S	S	-	-	-		
E209	2016	S	S	-	+	-		
E336	2018	S	S	-	+	-	296	
E189	2015	S	S	-	-	-	296	
E219	2016	S	S	-	-	-	296	
E193	2015	S	S	+	-	-		
E134	2014	S	S	-	-	-		
E375	2019	S	S	+	-	-		
E327	2018	S	S	+	-	-		
E16	2011	R	R	+	+	III	414	
E481	2020	R	R	+	+	-	17	
E191	2015	S	S	+	-	-		
E349	2019	R	R	+	+	-	17	Pulsotype 78
E362	2019	S	S	+	+	-	787	
E416	2019	S	S	+	-	III	80	
E04	2011	S	S	+	-	III	18	
E350	2019	S	S	+	-	-		
E403	2019	S	R	+	-	III	80	
E322	2018	S	R	+	-	III	80	
E24	2011	S	R	+	+	III	414	Pulsotype 79
E64	2012	R	S	+	-	III	18	
E06	2011	S	S	+	+	III		
E390	2019	S	R	+	+	IV	1984	Pulsotype 80
E422	2020	R	R	+	+	III	78	

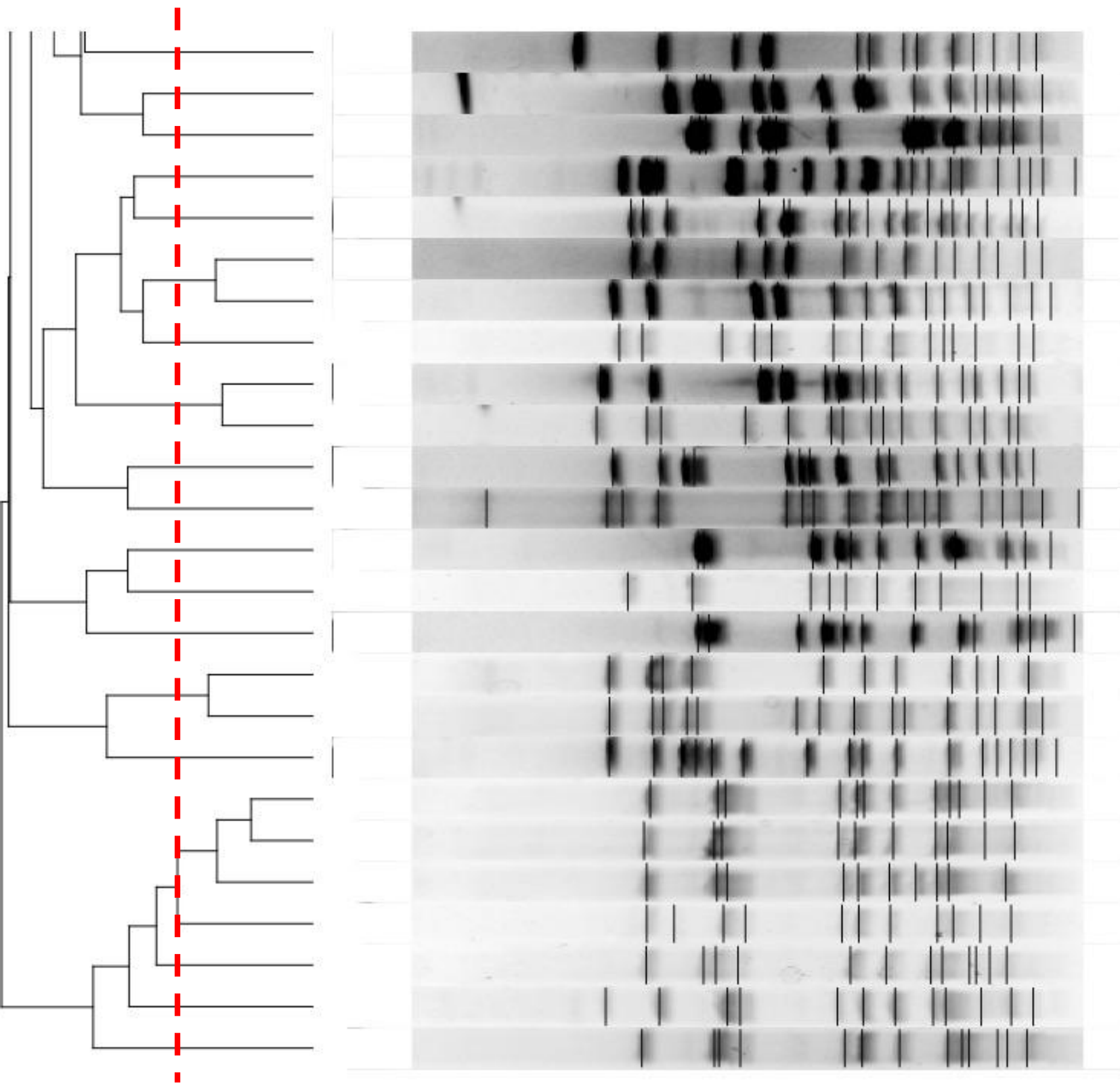

NO.	Year	Van	Gen	*repA_N*	*scrB*	Insert	ST	
E02	2011	S	R	+	+	III		
E58	2012	S	S	+	+	-		
E214	2016	S	S	+	-	III	80	
E39	2012	S	R	+	-	-		
E317	2018	S	S	+	+	-		
E10	2011	S	S	+	+	-		Pulsotype 81
E63	2012	S	S	+	+	-		
E412	2019	S	S	+	+	-		
E66	2012	S	S	+	-	-		Pulsotype 82
E190	2015	S	S	+	-	-		
E211	2016	S	S	+	-	-		
E371	2019	S	S	+	-	-		
E196	2015	S	S	+	+	-		
E418	2020	S	S	-	+	-		
E203	2016	S	S	-	-	-	54	
E463	2020	S	R	-	+	-		Pulsotype 83
E330	2018	S	S	+	-	-		
E174	2015	S	S	-	-	-		
E423	2020	S	S	+	+	III		Pulsotype 84
E427	2020	S	S	+	+	III	78	
E424	2020	R	S	+	+	III	78	
E419	2020	S	S	+	+	III	78	
E415	2019	S	S	+	+	III	78	
E451	2020	S	S	+	+	III	78	
E442	2020	S	S	+	+	III	78	

Fig. S2. PFGE analysis of SmaI-digested DNA of 465 *E. faecium* isolates in Taiwan, 2011 to 2020. Degree of similarity is shown in the scale. Red dashed line indicates 80% similarity. Vancomycin and gentamicin susceptibility testing are performed by agar dilution method. Presence of *repA_N* and *scrB*, as well as the types of IS*1216V* insertion *into aac(6')-Ie-aph(2")-Ia* are detected by PCR. ST of the selected isolates are determined by MLST. Abbreviations: Van, vancomycin; Gen, gentamicin; R, resistance; S, susceptible.
