# Supplementary material for: Widespread dissemination of the RepA_N-type megaplasmid conferring a carbohydrate utilization advantage in clonal complex 17 Enterococcus faecium in Taiwan
Source: Microbiol Spectr. 2026 Jul 14;14(8):e03793-25. doi: 10.1128/spectrum.03793-25 (PMC13436390; doi:10.1128/spectrum.03793-25)
Supplement: Supplementary file 3 [file spectrum.03793-25-s0003.pdf]

TABLE S1 Summary of gene clusters involved in the transport and utilization of carbohydrates or related compounds in ST17 *E. faecium* E37-21.

**Gene name**	**Product**	**Position**	**Related carbohydrate metabolism**
citG	2-(5''-triphosphoribosyl)-3'-dephosphocoenzyme-A synthase	14,534-15,397	
citX	Apo-citrate lyase phosphoribosyl-dephospho-CoA transferase	15,390-15,947	
citF	Citrate lyase alpha chain	15,944-17,503	
citE	Citrate lyase subunit beta	17,496-18,401	
citD	Citrate lyase acyl carrier protein	18,406-18,699	Citrate utilization gene cluster
citC	[Citrate [pro-3S]-lyase] ligase	18,703-19,254	
citC_2	Citrate lyase synthetase	19,251-19,715	
cimH	Citrate/malate transporter	19,741-21,045	
KPLKAHCH_00026	NAD-dependent malic enzyme	21,026-22,213	
lacX	Aldose 1-epimerase family protein	29,360-30,259	
KPLKAHCH_00039	Tagatose-bisphosphate aldolase	30,280-30,552	
lacG	6-phospho-beta-galactosidase	30,575-31,981	
lacE	PTS system lactose-specific EIICB component	32,123-33,829	
lacF	PTS system lactose-specific EIIA component	33,829-34,146	PTS-dependent lactose utilization gene cluster
lacD_1	Tagatose 1,6-diphosphate aldolase	34,174-35,154	
lacC	Tagatose-6-phosphate kinase	35,157-36,089	
lacB	Galactose-6-phosphate isomerase subunit LacB	36,100-36,615	
lacA	Galactose-6-phosphate isomerase subunit LacA	36.632-37,057	
lacR	Lactose phosphotransferase system repressor	37,547-38,314	
manR	Transcriptional regulator ManR, PTS transporter subunit EIIA	80,238-82,184	
KPLKAHCH_00094	PTS fructose transporter subunit IIA	82,187-82,642	
gatC	PTS system galactitol-specific EIIC component	82,656-83,984	PTS-dependent galactitol utilization gene cluster
KPLKAHCH_00096	PTS sugar transporter subunit IIB	84,018-84,302	
rpe	Ribulose-phosphate 3-epimerase	84,835-85,497	

hxlB	3-hexulose-6-phosphate isomerase	85,504-86,076	
gmuE	Putative fructokinase	95,484-96,356	PTS-dependent sucrose utilization gene cluster
KPLKAHCH_00114	Sucrose-specific PTS transporter subunit IIBC	97,966-99,912	
scrB	Sucrose-6-phosphate hydrolase	100,097-101,536	
degA	HTH-type transcriptional regulator DegA	101,538-102,500	
rpiR	HTH-type transcriptional regulator RpiR	107,362-108,180	PTS-dependent glucose and N-acetylmannosamine utilization gene cluster
nanE	Putative N-acetylmannosamine-6-phosphate 2-epimerase	108,341-109,030	
glcE	PTS system glucoside-specific EIICBA component	109,044-110,546	
KPLKAHCH_00126	PTS glucose transporter subunit IIA	110,559-111,035	
licC	Lichenan permease IIC component	118,684-119,934	Lichenan, tagatose, and D-galactosamine utilization gene cluster
lacD_2	Tagatose 1,6-diphosphate aldolase	120,958-121,953	
agaS	D-galactosamine-6-phosphate deaminase AgaS	121,969-123,138	
nagR	HTH-type transcriptional repressor NagR	123,154-123,888	
ngcG	Diacetylchitobiose uptake system permease protein NgcG	160,904-161,719	Gene cluster with diacetylchitobiose transport and glycolysis
gpmI	2,3-bisphosphoglycerate-independent phosphoglycerate mutase	161,716-162,540	

TABLE S2 Differences in regions between the pCC17-MCR in E198 and E250

Region	Location		Description
	E198	E250	
1	-	2420~3879	Additional IS*16* in E250
2	49,473~51,524	50,947~53,600	
3	-	97,791~97,979	Additional 189 nt non-coding sequence in E250
4	124,376~125,885	-	Additional IS*Efa11* in E198
5	173,148~174,928	-	Additional IS*Spn10* in E198
6	-	221,942~222,651	Additional intact *aac(6')-Ie-aph(2'')-Ia* in E250

TABLE S3 Summary of gene clusters involved in the transport and utilization of carbohydrates or related compounds in ST78 *E. faecium* E250.

Gene name	Product	Position	Related carbohydrate metabolism
yurK_1	Putative HTH-type transcriptional regulator YurK	56,688-57,422	PTS-dependent fructose, mannose, and sorbose transport gene cluster, and fructosamine utilization gene
frlB_1	Fructosamine deglycase FrlB	57,639-58,652	
levD_1	PTS system fructose-specific EIIA component	58,663-59,073	
AIOPLDIF_00065	Putative phosphotransferase enzyme IIB component	59,073-59,540	
sorA_1	PTS system sorbose-specific EIIC component	59,558-60,304	
manZ_1	PTS system mannose-specific EIID component	60,304-61,113	
levD_2	PTS system fructose-specific EIIA component	71,188-71,604	PTS-dependent fructose, mannose, and sorbose transport gene cluster, and fructosamine utilization gene
manX	PTS system mannose-specific EIIAB component	71,601-72,701	
sorA_2	PTS system sorbose-specific EIIC component	72,085-72,858	
manZ_2	PTS system mannose-specific EIID component	72,872-73,684	
frlB_2	Fructosamine deglycase FrlB	73,738-74,703	
norR	Anaerobic nitric oxide reductase transcription regulator NorR	74,840-77,563	
hxpB	Hexitol phosphatase B	77,576-78,208	
frlB_3	Fructosamine deglycase FrlB	85,263-86,231	Fructosamine utilization gene cluster
frlD	Fructosamine kinase FrlD	86,242-87,057	
yurK_2	Putative HTH-type transcriptional regulator YurK	87,125-87,835	
gmuE_1	Putative fructokinase	119,192-120,064	PTS-dependent sucrose utilization gene cluster
AIOPLDIF_00137	Sucrose-specific PTS transporter subunit IIBC	120,328-122,274	
scrB	Sucrose-6-phosphate hydrolase	122,459-123,898	
degA_1	HTH-type transcriptional regulator DegA	123,900-124,862	
rpiR	HTH-type transcriptional regulator RpiR	129,703-130,521	PTS-dependent glucose and N-acetylmannosamine utilization gene cluster
nanE	Putative N-acetylmannosamine-6-phosphate 2-epimerase	130,682-131,371	
glcB	PTS system glucoside-specific EIICBA component	131,385-132,887	
AIOPLDIF_00149	PTS glucose transporter subunit IIA	132,900-133,376	

gtfA	Sucrose phosphorylase	142,824-144,284	
malL	Oligo-1,6-glucosidase	144,281-145,501	
agaA_1	Alpha-galactosidase AgaA	145,562-147,745	
ngcG_1	Diacetylchitobiose uptake system permease protein NgcG	147,785-148,615	
melD_1	Melibiose/raffinose/stachyose import permease protein MelD	148,627-149,499	Gene cluster involved in the transport and
msmE	Multiple sugar-binding protein	149,509-150,768	utilization of diacetylchitobiose, melibiose,
melA	Alpha-galactosidase	150,916-152,226	raffinose, and stachyose
AIOPLDIF_00170	Carbohydrate ABC transporter permease	152,258-153,082	
melD_2	Melibiose/raffinose/stachyose import permease protein MelD	153,440-153,994	
AIOPLDIF_00172	Sugar ABC transporter substrate-binding protein	154,077-155,186	
melR	HTH-type transcriptional repressor MelR	155,480-156,505	
lacD	Tagatose 1,6-diphosphate aldolase	181,212-182,198	
lacC	Tagatose-6-phosphate kinase	182,211-183,137	
lacB	Galactose-6-phosphate isomerase subunit LacB	183,150-183,665	
lacA	Galactose-6-phosphate isomerase subunit LacA	183,684-184,112	
lacR_1	Lactose phosphotransferase system repressor	184,132-184,905	
AIOPLDIF_00205	PTS sugar transporter subunit IIA	185,070-185,549	
AIOPLDIF_00206	PTS fructose transporter subunit IIB	185,606-185,908	PTS-dependent lactose utilization gene
gatC	PTS galactitol transporter subunit IIC	185,930-187,387	cluster
lacR_2	Lactose phosphotransferase system repressor	187,980-188,732	
lacE	PTS system lactose-specific EIICB component	190,357-192,039	
lacF	PTS system lactose-specific EIIA component	192,050-192,373	
lacG_1	6-phospho-beta-galactosidase	193,396-193,850	
lacR_3	HTH-type transcriptional regulator LacR	194,269-195,276	
dgoA	2-dehydro-3-deoxy-6-phosphogalactonate aldolase	195,670-196,035	
gmuE_2	Putative fructokinase	202,911-203,783	

sacA	Sucrose-6-phosphate hydrolase	203,800-205,272	Gene cluster involved in lactose and L-arabinose transport and in the utilization of diacetylchitobiose and sucrose
lacG_2	Lactose transport system permease protein LacG	205,286-206,134	
araP	L-arabinose transport system permease protein AraP	206,131-207,015	
dasA	Diacetylchitobiose binding protein DasA	207,040-208,353	
degA_2	HTH-type transcriptional regulator DegA	208,493-209,464	
ngcG_2	Diacetylchitobiose uptake system permease protein NgcG	241,718-242,533	Gene cluster involved in diacetylchitobiose transport and glycolysis
gpmI	2,3-bisphosphoglycerate-independent phosphoglycerate mutase	242,530-243,354	
agaA_2	Alpha-galactosidase AgaA	262,357-264,606	Gene cluster involved in α-galactoside utilization
rhaR	HTH-type transcriptional activator RhaR	264,703-265,419	

Table S4 Major STs in *vanA*-positive VREfm, 2011 to 2020

Year	No. of *vanA*-positive VREfm	No. of isolates (%)		
		ST17	ST78	Others
2011	3	1 (33.3)	0 (0)	2 (66.7)
2012	2	0 (0)	0 (0)	2 (100.0)
2013	1	1 (100.0)	0 (0)	0 (0)
2014	1	0 (0)	1 (100.0)	0 (0)
2015	15	3 (20.0)	9 (60.0)	3 (20.0)
2016	16	1 (6.3)	12 (75.0)	3 (18.8)
2017	7	0 (0)	7 (100.0)	0 (0)
2018	29	9 (31.0)	20 (69.0)	0 (0)
2019	42	19 (45.2)	20 (47.6)	3 (7.1)
2020	38	19 (50.0)	13 (34.2)	6 (15.8)
Total	154	53 (34.4)	82 (53.2)	19 (12.3)

TABLE S5 Primers used in this study

Primer set	Target	Primer name	Sequence (5' to 3')
(i) Replication initiator gene			
I	*repA_N*	repA_N-F	ACACGACACTGTTAAAAGCC
		repA_N-R	CGTAATAGTGCTTGGGGAGC
(ii) Sucrose-6-phosphate hydrolase gene			
III	*scrB*	scrB-F	AAACGAGATAGCCACGGC
		scr-R	TCCTTGAGTCGCATACGC
(iii) Gentamicin resistance gene			
III	*aac(6')-Ie-aph(2'')-Ia*	aacA-aphD F	ATACAGAGCCTTGGGAAG
		aacA-aphD R	GCCACACTATCATAACC
(iv) Typing of IS*1216V* insertion types			
IV		aacA-aphD R	GCCACACTATCATAACC
		IS1216V 615-F	CGAAGTTTACGCACTGCC
(v) Detection of complete *aac(6')-Ie-aph(2'')-Ia*			
V	Tn*5281*	Tn5281-F	GGACTTGACTCACTTCCT
		Tn5281-R	ATGTATATGAAAAATTAGGTGG

Presence of IS*1216V* -inserted *aac(6')-Ie-aph(2'')-Ia* was determiend by primer sets III and IV.
